# Fatty Acid Metabolism and T Cells in Multiple Sclerosis

**DOI:** 10.3389/fimmu.2022.869197

**Published:** 2022-05-04

**Authors:** Saige L. Pompura, David A. Hafler, Margarita Dominguez-Villar

**Affiliations:** ^1^ Departments of Neurology and Immunobiology, Yale School of Medicine, New Haven, CT, United States; ^2^ Faculty of Medicine, Imperial College London, London, United Kingdom

**Keywords:** T cell, multiple sclerosis, immunometabolism, fatty acids, T regulatory (Treg) cell

## Abstract

Cellular metabolic remodeling is intrinsically linked to the development, activation, differentiation, function, and survival of T cells. T cells transition from a catabolic, naïve state to an anabolic effector state upon T cell activation. Subsequently, specialization of T cells into T helper (Th) subsets, including regulatory T cells (T_reg_), requires fine-tuning of metabolic programs that better support and optimize T cell functions for that particular environment. Increasingly, studies have shown that changes in nutrient availability at both the cellular and organismal level during disease states can alter T cell function, highlighting the importance of better characterizing metabolic-immune axes in both physiological and disease settings. In support of these data, a growing body of evidence is emerging that shows specific lipid species are capable of altering the inflammatory functional phenotypes of T cells. In this review we summarize the metabolic programs shown to support naïve and effector T cells, and those driving Th subsets. We then discuss changes to lipid profiles in patients with multiple sclerosis, and focus on how the presence of specific lipid species can alter cellular metabolism and function of T cells.

## Introduction

During the course of an immune response, CD4^+^ T cells transition from a naïve state, participating in immune surveillance, to a highly specialized subset of effector T cells (T_eff_) equipped with the capacity to rid hosts of invading pathogens and regulate immune responses. As this transition occurs, there is concurrent cellular metabolic remodeling that is intrinsically linked to the development, activation, differentiation, function, and survival of T cells. In addition to the different metabolic requirements of naïve and effector T cells, there are additional metabolic dependencies for CD4^+^ T helper (Th) subsets and regulatory T cells (T_regs_), the latter relying on a different metabolic program than their effector counterparts, displaying metabolic flexibility while maintaining suppressive functions. For example, memory CD8^+^ T cells use fatty acids derived from extracellular glucose or those mobilized from lysosomes to support their fatty acid oxidation (FAO), whereas T_regs_ acquire fatty acids from the extracellular environment, although the mechanism is not well understood (1).

At the cellular and organismal level, previous studies have shown that nutrient availability in physiological health and disease states can alter T cell function. For example, fluctuations in salt intake and lipid composition in the diet have been shown to change the inflammatory profile of T cells (2–4), and feeding mice diets containing different fatty acid species alter the ratios of T cell subsets and outcome of experimental autoimmune encephalomyelitis (EAE) (5). Additionally, at the cellular level, manipulating metabolic pathways in T cells skews their differentiation towards specific Th cell subsets. Depending on the specific context this can be harmful or beneficial, as metabolic manipulation often determines Th17 versus T_reg_ cell fates (6, 7). Together, the importance of the metabolic-immune axis is critical. However, while the link between metabolism and T cell function is appreciated, specifically how fluctuations in lipid availability, lipid species, and lipid metabolism dictates T cell functions and phenotypes is not well investigated. Studies have also shown that there are changes to lipid profiles in inflammatory settings such as obesity and autoimmune diseases like multiple sclerosis (MS) (8–10), further highlighting the importance of teasing apart the intricate links between metabolic and transcriptional changes within T cells.

In this review we discuss the changes in metabolic states as T cells change from naïve to effector cells and discuss the metabolic requirements driving CD4^+^ T cell subsets, including T_reg_ cells. We briefly highlight the key roles of T cells in the pathogenesis of multiple sclerosis, and detail findings of altered lipid profiles in MS and murine models of EAE. We highlight data showing that altering lipid metabolism in T cells can alter T cell function in MS and EAE. Finally, we summarize how lipids are trafficked in the body and provide evidence supporting the idea that specific lipid species are capable of altering T cell phenotypes and functions.

## Immunometabolism of T Cells

### Metabolic Remodeling of T Cells Upon Activation

Upon activation, quiescent, naïve T cells have an increased need for cellular energy in the form of adenosine triphosphate (ATP) and biomass to meet proliferative demands and production of effector molecules. As such, the rapidly dividing cells utilize aerobic glycolysis, by which pyruvate is used to produce lactate in the presence of oxygen by cells with a great enough mitochondrial capacity to perform oxidative phosphorylation (OXPHOS), known as the Warburg effect (11, 12). T cell receptor (TCR) signals induce c-Myc, which initiates the expression of metabolic genes to induce glycolysis and mitochondrial metabolism necessary for T cell activation (13, 14). Both c-Myc and mammalian target of rapamycin (mTOR) are positive regulators of glycolysis in T cells (13, 15). mTOR exists as two complexes, mTORC1 and mTORC2. mTORC1 enhances glycolytic metabolism and can regulate other metabolic programs such as lipid synthesis and amino acid metabolism (16). mTOR can partially mediate TCR sensitivity (17), and co-stimulatory signals from CD28 have been shown to upregulate glucose utilization in T cells by recruitment of the phosphatidylinositol 3-kinase (PI3K) catalytic subunit p110γ which activates AKT and mTORC1 (18, 19).

**Figure 1:**
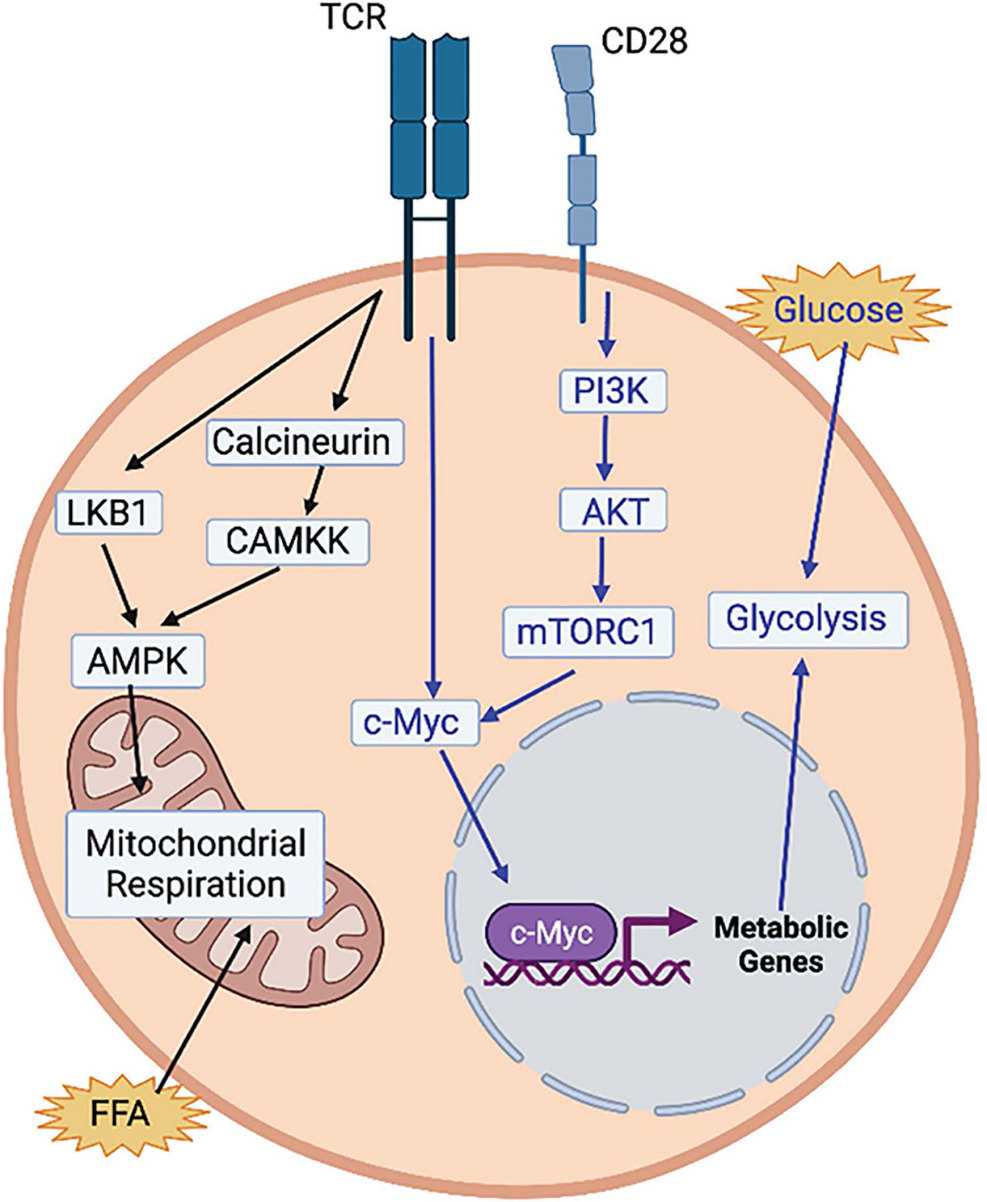
Regulation of T cell activation by metabolic pathways. T cell receptor (TCR) activation activates AMPK via 2 pathways (black arrows). Via an LKB1-dependent pathway and via and LKB1-independent pathway, through calcineurin and CAMKK. Both AMPK and uptake of free fatty acids (FFA) activate mitochondrial respiration, which has been shown to be necessary for T cell activation. Activation of glycolysis is also essential for T cell activation (blue arrows). TCR activation activates c-Myc which turns on the expression of glycolytic metabolic genes. CD28 activation also activates c-Myc via the PI3K-AKT-mTORC1 pathway. Image created with BioRender.com

AMP-activated protein kinase (AMPK) is another central node controlling metabolic remodeling during T cell activation. It is activated by TCR signaling *via* two different pathways. The first pathway is LKB1-dependent: AMPK can sense the cellular AMP-to-ATP ratio and control the progression of cells to complete activation (20). The second pathway of AMPK activation is calcium-dependent, mediated by activation of calcineurin and downstream kinase CAMKK (21) (**Figure 1**). T cells that lack AMPK are completely dependent on glycolysis and do not re-engage mitochondrial respiration upon glucose depletion (22). This suggests that activation of AMPK in early T cell activation limits mTOR signaling and prevents premature engagement of glycolysis that is associated with the high proliferative demands of clonal expansion (23). In the mitochondria, the tricarboxylic acid (TCA) cycle creates reducing equivalents that are fed into the electron transport chain (ETC) to generate ATP *via* OXPHOS. Substrates are derived from multiple cytosolic and mitochondrial metabolic pathways including glycolysis and the oxidation of amino acids and fatty acids. FAO translocates free fatty acids (FFA) into the mitochondrial matrix to be broken down and produce acetyl-CoA, which enters the TCA. Acetyl-CoA can also act as a substrate for epigenetic modifications, thereby illustrating how metabolism can directly influence gene expression (24). During the first 24-48 hours after T cell activation, T cells are dependent on ATP production from ATP synthase to become fully activated and meet proliferative demands (25). This reliance on ATP is demonstrated by limited T cell activation after knockdown of the ETC complex IV subunit COX10 (26), and by the inhibition of antigen-specific T cell activation upon loss of ETC complex III subunit RISP (27).

### Metabolic Requirements of CD4^+^ T Cell Subsets

**Figure 2:**
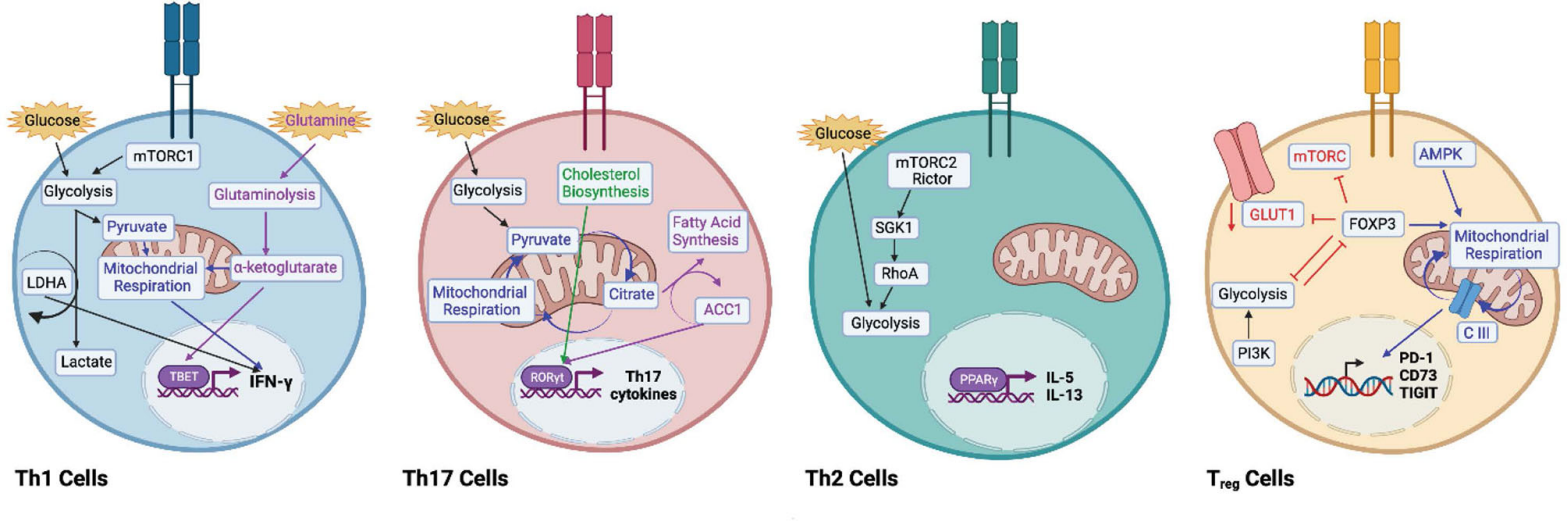
Metabolic remodelling to promote Th helper T cell subsets. Th1 cells engage in glycolysis (black arrows) and aerobic respiration (blue arrows) to support interferon (IFN)-γ expression. Lactate dehydrogenase (LDHA) has been shown to promote IFN-γ expression. Inhibition of mTORC1 decreases IFN-γ expression. Glutaminolysis (purple arrows) via ⍺-ketoglutarate promotes TBET expression. Th17 cells use both glycolysis (black arrows) and oxidative phosphorylation (blue arrows) to secrete Th17-associated cytokines. Acetyl-CoA carboxylase 1 (ACC1) promotes RORγt expression (purple arrows), and intermediates of cholesterol biosynthesis (green arrows) support the RORγt program. Th2 cells are supported by high levels of glycolysis. The mTORC2-Rictor complex supports the Th2 lineage via SGK1 and RhoA (black arrows). Peroxisome proliferator- activated receptor (PPAR)-γ has been shown to promote IL-5 and IL-13. Regulatory T (T_reg_) cells are supported by mitochondrial respiration (blue arrows), as AMPK expression drives fatty acid oxidation and oxidative phosphorylation. Complex III of the electron transport chain supports the expression of immunosuppressive genes like PD-1, CD73, and TIGIT. Expression of FOXP3 promotes AMPK expression and inhibits expression of mTORC1, GLUT1, and glycolysis (red arrows). Activation of PI3K drives glycolysis and decreases Treg suppression (black arrows). Glycolysis and FOXP3 reciprocally regulate each other. Image created with BioRender.com

After the initial metabolic reprogramming that occurs with T cell activation, T_eff_ cells use glycolysis to proliferate and maintain their effector functions (28–30). However, Th cell subsets are supported by unique metabolic needs. For example, Th1 cells rely on glycolysis and glutaminolysis to support their growth and proliferation (31), and activating naïve CD4^+^ T cells in a glutamine deficient environment generates FOXP3^+^ T_regs_ even in the presence of Th1 polarizing cytokines (32). Further, the by-product of glutaminolysis, α-ketoglutarate, might serve as the metabolic determinant in Th1 differentiation by promoting expression of TBET and mTORC1 signaling (32). mTORC1 has been shown to phosphorylate TBET, and inhibition of mTORC1 signaling reduces production of interferon gamma (IFN)-γ (33), highlighting the importance of glucose uptake and aerobic glycolysis in the production of IFN-γ (25, 34, 35). Th1 expression of IFN-γ production is regulated transcriptionally and post-transcriptionally (22, 25), and when activated under glucose-depleted conditions, production of IFN-γ is inhibited (25, 35–38). IFN-γ production is limited by the binding of GAPDH to the 3’ UTR of *ifng* mRNA, which is reversed when GAPDH is engaged in glycolysis (25). Epigenetically, glycolysis has been shown to promote IFN-γ production in Th1 cells *via* lactate dehydrogenase (LDHA) (35). LDHA is required to sustain aerobic glycolysis that supports Th1 differentiation, and loss of LDHA leads to reduced H3K9 acetylation at the *ifng* locus that is associated with active transcription and reduced glycolytic flux (35) (**Figure 2**). Moreover, a recent study has demonstrated a role for mitochondrial metabolism in Th1 effector function by showing that mitochondrial metabolism uncouples Th1 proliferation and effector functions. The TCA cycle, and specifically, succinate dehydrogenase activity (ETC complex II), is required for Th1 effector functions including expression of IFN-γ, but suppresses proliferation and histone acetylation. Conversely, complex I of the ETC, the malate-aspartate shuttle, and mitochondrial citrate export maintain Th1 proliferation and promote histone acetylation to regulate T cell activation genes (39).

In correlation with enhanced glycolytic metabolism, *in vitro*-generated Th17 cells express higher levels of pyruvate dehydrogenase kinase 1 (PDK1), which is essential for Th17 differentiation *in vitro* (40, 41). Th17 cells differentiated *in vitro* utilize both OXPHOS and glycolysis, and Th17 cells from steady state and inflamed tissues rely on OXPHOS for cytokine production (40). In support of these data, the OXPHOS inhibitor oligomycin reduces Th17 pro-inflammatory cytokine production and improves pathology in mouse models of colitis (40). Evidence suggests that lipid metabolism plays an important role in the function of Th17 cells under normal and stressed conditions (42, 43), and Th17 cells rely on *de novo* fatty acid synthesis, rather than acquisition of extracellular fatty acids (42). While the reasons for this are unclear, it has been shown that Th17 cells are particularly dependent on cholesterol synthesis, as intermediates of the pathway can bind to the RORγt promoter and promote interleukin (IL)-17 expression, whereas inhibition of cholesterol biosynthesis blocks Th17 development (44, 45). The ratio of intracellular saturated to polyunsaturated fatty acids has been shown to control CD5L expression, which can determine whether or not Th17 cells will adopt a pro- or anti-inflammatory phenotype (46). Saturated to polyunsaturated FFA ratios also affect the expression of metabolic genes *cyp51* and *sc4mol*, which synthesize ligands for RORγt (43, 46). Given the intricacies of Th17 and T_reg_ development, studies are unveiling a role for metabolic intermediates in skewing T cell lineages alongside cytokine polarization. For example, acetyl-CoA carboxylase-1 (ACC1)-deficient T cells default to the T_reg_ lineage (6), and mice fed a high fat diet increases the expression of ACC1 in CD4^+^ T cells, which upregulates the expression of the Th17 master regulator, RORγt (7) (**Figure 2**). The increase of ACC1 expression in CD4^+^ T cells is mimicked in obese individuals and proposed to contribute to associated Th17 pathologies (7).

Th2 cells have been reported to display high glycolytic activity (31, 47). Ras homologue enriched in brain (Rheb) is a small GTPase that activates mTORC1. Rheb-deficient mice have impaired mTORC1 function and fail to generate Th1 and Th17 cells without alterations in Th2 differentiation. Conversely, rapamycin-insensitive companion of TOR (Rictor) is a component of the mTORC2 complex, and Rictor-deficient mice have impaired mTORC2 signaling, with differentiation into Th2 cells, but intact Th1 and Th17 generation (48). Further, downstream targets of mTORC2, SGK1 and the GTPase RhoA, have been shown to play important roles in the commitment to, and function of Th2 cells, respectively (49, 50). These data suggest mTORC2 is the signaling determinant of the Th2 lineage, not mTORC1, and that glycolytic engagement differs between Th2 and Th1 and Th17 lineages. Genome-wide transcriptional profiling of human Th2 cells from allergic asthma patients demonstrate a positive correlation between the expression of c-Myc and disease state further supporting glycolysis as a marker of Th2 pathogenicity (51). Lipids might also play a role in Th2 metabolism, as expression of peroxisome proliferator-activated receptor (PPAR)-γ is critical for Th2 responses against house dust mite antigens, and PPARγ-deficient mice display Th2 cells unable to produce IL-5 and IL-13 (51, 52) (**Figure 2**).

### Metabolic Adaptations of Regulatory T Cells

In contrast to T_eff_ cells, T_regs_ appear to exhibit a greater capacity for metabolic flexibility. Murine T_regs_ have been described to preferentially rely on OXPHOS rather than aerobic glycolysis for ATP production (31). However, metabolic adaptations of T_reg_ cells are context-dependent and influenced by T_reg_ origin and anatomical distribution (53). It remains unclear how thymic-derived T_regs_ (tT_regs_) and T_regs_ differentiated in the periphery from conventional T_eff_ cells, termed peripheral T_regs_ (pT_regs_) differ metabolically. However, it appears that T_reg_ proliferation and effector functions are maintained through different metabolic programs. Proteomic analysis of human T_reg_ and T_eff_ cells suggests that freshly isolated T_regs_ are highly glycolytic and proliferative, while *in vitro* proliferating T_regs_ engage in both glycolysis and FAO (53). Conversely, T_eff_ cells switch from OXPHOS to aerobic glycolysis upon *in vitro* activation, and FAO appears to be indispensable for proliferation and effector functions (53). One explanation for the discrepancy between human and murine models is that mice are typically housed in pathogen-free environments, whereas human immune cells are constantly engaging with pathogens that requires T_regs_ to meet higher proliferative and functional demands (54).

AMPK and mTORC1 signals are important determinants of T_reg_ fitness. T_reg_-specific mTOR deletion impairs T_reg_ fitness and homeostasis resulting in a reduction of T_regs_ in the tissues and T_eff_ cell activation and heightened inflammation at barrier sites (55). Inhibiting mTORC1 by rapamycin along with IL-2 signaling greatly expands T_reg_ numbers (56). Conversely, AMPK, which activates FAO and OXPHOS, provides critical signals required for T_reg_ differentiation (57). Activation of AMPK by metformin induces T_reg_ differentiation and, in murine models, was sufficient to limit progression of EAE and inflammatory bowel disease (31, 58, 59).

FOXP3 is involved in the fine tuning of metabolic cues to maintain T_reg_ function. Inflammatory stimuli and FOXP3 oppose each other through regulation of mTORC1 and glucose metabolism as T_reg_ treatment with TLR1/TLR2 agonists enhances mTORC1 activity and T_reg_ proliferation but reduces their suppressive capacity (60). In contrast, FOXP3 inhibits mTORC1 signaling and glycolysis while promoting OXPHOS and reduces the rate of proliferation (60), suggesting that mTORC1 activity and glycolysis are adequate for T_reg_ proliferation in inflammatory environments. However, excessive mTORC1 activity impairs T_reg_ function and indeed T_regs_ suppress mTORC1 activity *via* PP2A (48, 61, 62). Murine T_regs_ induced *via* transforming growth factor (TGF)-β express low levels of GLUT1 compared to T_eff_ cells (31), and in human T_regs_, GLUT1 expression is also thought to be limited by FOXP3 *via* AKT inhibition (63). T_regs_ that overexpress GLUT1 exhibit reduced CD25 and FOXP3 expression and fail to suppress colitis (60). Expression of FOXP3 allows T_regs_ to survive in lactate rich environments by increasing the ratio of NAD to NADH (64). T_reg_ proliferation and suppressive functions can be uncoupled metabolically, which is likely advantageous in an inflammatory setting. For example, it might be advantageous for T_regs_ to engage in glycolysis to rapidly proliferate and deprive proliferating T_eff_ cells of an essential nutrients, then switch to an OXPHOS program for optimal suppressor functions. Of note, PI3K-mTORC2 mediated activation of glucokinase, which activates glycolysis and mediates actin cytoskeletal rearrangements, was found to be important for proper migration of T_regs_ in skin allograft models (65). Therefore, although glycolysis seems to play a role in T_reg_ migration and proliferation, utilization of OXPHOS appears to be the main metabolic program utilized for T_reg_ function, which is dependent on FAO (31).

Cytokines that promote T_reg_ differentiation such as TGF-β (66), activate AMPK (67) and promote FAO to skew naïve T cells into a T_reg_ phenotype instead of pro-inflammatory Th17 cells (31, 68). In support of this observation, T_reg_ differentiation and suppressive functions are reduced by the FAO inhibitor etomoxir (31), and suppression of PI3K activity by phosphatase and tensin homolog (PTEN) drives T_reg_ differentiation (69). PTEN-deficient T_regs_ have elevated glycolytic activity and lose FOXP3 expression and suppressive function (70). These data suggest that the balance between PTEN and AKT functions can regulate T_reg_ differentiation. Data also suggest a reciprocal relationship between FOXP3 and glycolysis as FOXP3 has been shown to interfere with c-Myc expression to dampen glycolytic activity (64) and forced expression of FOXP3 suppresses glycolysis-related genes while inducing lipid and oxidative metabolic genes required for maximum suppression (60). In murine models, mitochondrial complex III is required for T_reg_ suppression through the active metabolism of 2-HG and succinate, resulting in de-repressed transcription of T_reg_ suppressive genes, including PD-1, CD73, and TIGIT (**Figure 2**). Moreover, loss of mitochondrial complex III function accumulates 2-HG and succinate, which inhibits demethylases and represses transcription of T_reg_ suppressive genes PD-1, CD73, Nrp1, and TIGIT. In addition, mice containing a RISP knockout, an essential component of mitochondrial complex III, die after one month exhibiting lethal inflammation as characterized by enlargement of lymph nodes and spleens along with lymphocytic infiltration into multiple organs (71).

Interestingly, in human T_regs_, glycolytic activity has been linked to suppressive function *via* enolase-1, which mediates expression of a specific splice variant of FOXP3 that is necessary for suppression (72). However, this study also showed that the relationship between FOXP3 and metabolic intermediates changes depending on which metabolic program the cells are engaging. Upon inhibition of glycolysis with 2-DG, the non-glycolytic activity of enolase-1 represses FOXP3 expression, whereas the generation of T_regs_ under suboptimal TCR stimulation promotes FOXP3 expression *via* the glycolytic activity of enolase-1 (72) (**Figure 2**). Lack of mTOR activity does not decrease FOXP3 expression, but it does diminish cholesterol biosynthesis that has been shown to be required for suppressive function *in vivo* (73). Therefore, T_regs_ seem to be partially dependent on mTOR and glycolytic signals that are more oscillatory in nature (74), or perhaps for maintenance of metabolic intermediates that feed into lipid related pathways, as seen with cholesterol.

Fatty acids can induce cell death in T cells (75–78). It is suggested that T_regs_ have specifically evolved FAO dependence to combat fatty-acid-induced cell death *via* FOXP3 (79). For example, long chain fatty acids like palmitate, are pro-apoptotic through various mechanisms, including depolarization of mitochondrial action potential and reactive oxygen species generation (80). This shows that FOXP3 uniquely enables T_regs_ to utilize fatty acids as fuel by upregulating enzymes to engage in FAO and become resistant to apoptosis (79). This evolved anti-apoptotic strategy is likely even more useful during steady state or inflammation as T_regs_ are important in the maintenance of homeostasis in lipid-rich tissue environments. Undoubtedly, the metabolic-functional axis in these cells is incredibly complex, and is essential to our complete understanding of disease pathology and resolution.

Overall, it is clear that T cell metabolic programs are flexible and highly dynamic. T cells can utilize different substrates based on metabolic needs, and tailor metabolic programs to support effector functions. Studies investigating Th-subset differentiation show that specific metabolic engagements promote and suppress specific Th-lineage differentiation and functions.

## The Role of Lipids in T Cell Function

### Introduction to Lipids and Lipid Trafficking

Lipids provide the foundation of both cellular and organelle membranes, serve as fuel for cells, and are the precursors of bioactive lipid mediators. Lipids are incredibly diverse, just like proteins; however, because of limitations in technologies to study lipids, we are just beginning to understand the diverse effects lipids exert on biological processes such as gene expression and cellular function. It is understood that the storage and secretion of lipids from the adipose tissue is one mechanism by which the body can communicate hunger and the need for energy (81, 82). However, during lipolysis and even inflammation, FFAs are exposed to immune cells that reside in the tissues and in circulation. How immune cells sense and interpret these signals remains an active area of research, as evidence is emerging that not all lipids are created equal and perform the same functions on specific cell types.

Cells obtain fatty acids from lipid species in the diet, fats stored in cells as lipid droplets, and lipids synthesized in one organ for export to another. Lipid species from the diet are ingested as triglycerides, which travel into the small intestine where they are emulsified by bile salts, and form hydrophobic structures called micelles. Together, bile salts and pancreatic lipases breakdown triglycerides into FFAs. FFAs in the intestine are transported across intestinal cell membranes, where they are converted back into triglycerides, and packaged with cholesterol and other apolipoproteins into vesicles called chylomicrons. Chylomicrons move freely from the intestines, through the lymphatic system, and into the bloodstream where they either enter the liver, or the adipose tissue. In the adipose, they will either be oxidized for fuel, or stored until they are needed. When hormones in the body signal the need for metabolic fuel, triglycerides that are stored in adipose tissue are mobilized and transported to other tissues throughout the body where the fatty acids may be oxidized for energy (81).

Similar to amino acids, there are both essential and non-essential fatty acids. Essential fatty acids comprise groups of lipids that must be acquired from the diet, as they cannot be synthesized in the body. These include omega-3 and omega-6 fatty acids. Omega-3 fats are derived from fish, eggs, and other plant based sources and are typically associated with anti-inflammatory effects (82). In contrast, omega-6 fatty acids are found various oils and animal meats and are associated with the production of inflammatory bioactive mediators (83). Of interest, these two groups of fatty acids can compete with each other for insertion into cellular membranes, where they are cleaved and converted into lipid mediators and other signaling molecules (84). Further, studies have shown due to this competition, fats in the diet reflect the overall lipid composition in cellular membranes (84–87), highlighting an important link between diet and inflammatory status. Therefore, fat sources enriched in the Westernized diet may partially explain rises in some inflammatory associated diseases.

FFAs are further characterized according to length and saturation. SCFA are typically classified as having fewer than six carbons, whereas medium chain fatty acids (MCFA) contain between six to twelve carbons, and LCFA are those with more than twelve carbons. Saturated fatty acids contain no double bonds in their carbon tail, whereas monounsaturated and polyunsaturated fatty acids contain one double bond, and more than one double bond, respectively. SCFAs are able to freely move into the mitochondria for β-oxidation, whereas LCFAs must first enter the carnitine shuttle. Of note, the majority of FFAs obtained from the diet or released from the adipose tissue are MCFA or LCFAs. On the other hand, most of the SCFA studied are those derived from bacterial metabolism and associated byproducts.

### Lipids Altering Immune Cell Phenotypes

There is great focus on understanding factors that drive and regulate immune cell phenotypes and functions in tissues. Recent studies have described contributions from cytokines and hormones secreted from tissue-resident cells and other neighboring immune cells (88–91), the presence of local antigens driving immune cell persistence in the tissues (88, 92, 93), and the changes in nutrients that prompt metabolic reprogramming in specific microenvironments, such as tumors (94, 95). Evidence has shown that CD4^+^ T cell fate is driven by availability of metabolic substrates and cell-intrinsic programs that determine metabolic requirements (96). Metabolic reprogramming is critical for T cell proliferation, differentiation, and function, however, how specific substrates such as lipids, affect T cells remains unanswered. T_regs_ rely mainly on FAO-driven OXPHOS for survival and function (31), so lipid species in the tissues likely provide a critical signal for maintenance of T_reg_ survival and homeostatic functions within tissues as well. It is likely that altered lipid profiles in disease settings can impact T cell function and is of great interest to fully understand how tissue microenvironments shape T cell metabolism.

Recent data have shown that the length of fatty acids can differentially affect immune cell phenotypes. In the colon, resident gut bacteria produce SCFAs as a byproduct of fermentation (97, 98), which shapes the colonic T_reg_ population (99). The addition of the SCFA propionate to the drinking water of germ-free mice increases T_reg_ numbers in the colon, but not spleen or thymic T_reg_ numbers. Further, antibiotic-mediated depletion of gut bacteria reduces T_reg_ numbers that can be rescued with the addition of propionate to the drinking water (99). SCFAs promote FOXP3 expression and are dependent on fatty acid receptor, GPR43 (100, 101). In support of these data, the addition of SCFAs to naïve CD4^+^ T cells increases the proportion of FOXP3^+^ cells upon activation. However, the addition of LCFAs results in CD4^+^ T cells that produce more pro-inflammatory cytokines including IFN-γ and IL-17a that exacerbate EAE (5).

Unsaturated fatty acids like oleic acid and linoleic acid have been shown to modulate cytokine production in T cells (102, 103), but only saturated fatty acids induce cytokine secretion in the absence of T cell activation in a dose-dependent manner (102). In T cells, oleic acid has been shown to induce proliferation in the spleen and lymph nodes, but to inhibit Jurkat cell proliferation and IL-2 and IFN-γ production (104). In CD4^+^ T cells, fatty acid synthesis blockade by the inhibitor TOFA, reduces proliferation, which can be rescued by the addition of oleic acid specifically (105). Moreover, oleic acid can also rescue proliferation of CD4^+^ T cells cultured in fatty acid-free media (105). Interestingly, oleic acid has also been shown to decrease the content of arachidonic acid in cell membranes that result in decreased arachidonic acid-derived pro-inflammatory lipid mediators (86, 87), and to inhibit palmitic-induced inflammation in type 2 diabetes (106). Mechanistically, attenuation of palmitic acid-induced inflammation by oleic acid is linked to increases in CPT1a expression and FAO driven by AMPK activation. In adipocytes, oleic acid induces IL-10 and adiponectin expression which can activate AMPK (106). We have also shown that oleic acid is one of the most prevalent fatty acids in human adipose tissue, and is significantly decreased in the adipose tissue from MS patients (4).

In EAE models, docosahexaenoic acid (DHA) downregulates Th1 and Th17-related cytokines (107). Both DHA and eicosapentaenoic acid (EPA) have been shown to modulate the JAK-STAT pathway and IL-2 signaling in T cells (108). In Jurkat cell lines DHA exerts immunosuppressive effects by increasing calcium concentrations in cells (109–111). Similarly *in vivo*, mice fed a DHA- and EPA-enriched diet exhibited reduced T cell proliferation and IL-2 signaling (77, 112, 113). DHA has been also shown to induce dose-dependent reductions in the ability of T_regs_ to inhibit effector T cell proliferation. In contrast, DHA can upregulate FOXP3 mRNA and other immunosuppressive cytokines such as IL-10 (114).

Saturated fatty acids like palmitic acid have been shown to drive T cell activation *via* the PI3K/AKT pathway (5, 115, 116). Palmitic acid treatment also induces the expression of cytokines related to T cell activation such as IL-2, IL-6, IL-17A, TNF-α, and IFN-γ (102, 116). Similarly, lauric acid has been shown to have some pro-inflammatory properties, as it increases IL-2 and can promote Th17 differentiation in models of EAE (117).

The PPAR family of lipid receptors has been implicated in T cell biology, through regulation of IL-2 production (118–120), influencing Th17 and T_reg_ differentiation (121), and regulation of inflammation (121–126). Similarly, SREBPs are transcription factors that regulate gene expression related to lipogenesis and cholesterol synthesis and uptake (127). SREBPs are important for lipid membrane synthesis that allows rapid expansion of proliferating T cells (128). However, in the absence of fatty acids or upon blockade of FAO, T_reg_ development is inhibited *in vitro.* T_regs_ rely on exogenous fatty acids as their metabolic substrate (31, 42), which seems to impart a survival advantage for T_regs_ as FOXP3 alone upregulates enzymes associated with FAO and OXPHOS (79). Interestingly, triglyceride storage in T_regs_ limits protein kinase C activity to promote FOXP3 expression, potentially highlighting a feedback mechanism between exogenous fatty acid uptake in T_regs_ (79). Much of these data support the idea that fatty acids can differentially effect T_eff_ cells and T_reg_ subsets, but further, that specific fatty acid species have the potential to differentially affect T_reg_ biology. However, the mechanism driving these changes remains unknown.

## T Cells in Multiple Sclerosis

MS is an autoimmune disorder characterized by aberrant immune responses and immune cell infiltration in the central nervous system (CNS). Both relapsing-remitting MS and progressive MS experience demyelination and neurodegeneration because of ongoing inflammation in the CNS (129–131). Multiple immune cell types are found in the MS brain; however, autoreactive T cells are considered the main mediators of inflammation (132–137). In particular, the CD4^+^ T cell subsets, Th1 and Th17 cells promote inflammation *via* interactions with other immune cells types, such as B cells, follicular T helper cells (T_fh_) cells, and resident CNS microglia. T_fh_ cells support B cell survival, differentiation, and expansion *via* interactions in the germinal center and through secretion of cytokines like IL-21 (138, 139). T_fh_ cells have been reported in MS lesions (140). In addition, numerous markers of T_fh_ cells, such as IL-21, are upregulated in MS (141), and altered ratios of T_fh_ cells have been reported (142). For instance, MS patients have elevated ratios of Th17-like T_fh_ cells, which are considered pathogenic and support B cell antibody production (142). Thus, T_fh_ cells likely play an important role in MS pathogenesis by supporting autoreactive B cell antibody production.

Th1 CD4^+^ T cells that are associated with MS express the transcription factor TBET and secrete pro-inflammatory cytokines IFN-γ and TNF-α, whereas Th17 associated CD4^+^ T cells express RORC2 and secrete IL-17, IL-21, and IL-22 cytokines. Both IFN-γ and IL-17 are thought to enhance immune activation and have been strongly associated in human disease, as the frequencies of Th1 and Th17 cells are increased in MS lesions and the cerebrospinal fluid (CSF) (130, 143). However, there is a dichotomous nature to the roles of Th1 and Th17 cells in MS. Increases in Th17 cells have been measured during clinical relapse in the CSF of MS patients (143); whereas other studies have found increases in IFN-γ upon relapse (144). Despite these differences, evidence suggests that both IFN-γ and IL-17 are pathogenic in MS, however, different cytokines might play different roles depending on the stage of the disease (for example initiating events of the disease versus exacerbation of the disease). Furthermore, with advances in single cell technologies, there is growing evidence of subsets within Th cell subsets, so it is likely that not all IFN-γ or IL-17-producing cells are pathogenic, but perhaps only a subset of these cells, making the signals and environmental cues that drive these pathogenic subsets of great interest to define.

Of importance are the pathogenic Th17 cells that secrete both IFN-γ and IL-17 and express both TBET and RORγt, termed Th1-like Th17 cells, which are found in multiple autoimmune disorders like multiple sclerosis (145), insulin-dependent diabetes mellitus, autoimmune arthritis and inflammatory colitis (146, 147). In EAE, Th1-like Th17 cells have been found to be the main drivers of disease pathology (148), and in humans, IFN-γ^+^;IL-17^+^ T cells are found in the blood and brain tissue of MS patients (149). These Th1-like Th17 cells are capable of crossing the blood brain barrier and have been correlated with inflammation in both MS patients and EAE models (148–150). Th1-like Th17 cells can also secrete granulocyte macrophage colony-stimulating factor (GM-CSF) which augments inflammation by activation of innate immune cell populations (151, 152). In contrast, subsets of Th17 cells can produce IL-10 and thought to play a more suppressive, protective role in inflammatory settings (153–156).

Another consequence of the ongoing inflammation in MS are the functional changes that occur in T_regs_. Under homeostatic conditions T_regs_ are responsible for the maintenance of immune tolerance by preventing aberrant or excessive immune responses. However, during chronic inflammation, as with MS, T_regs_ acquire effector like properties, and express IFN-γ, termed, Th1-like T_regs_ (157). Th1-like T_regs_ upregulate other Th1 markers, such as TBET and CXCR3, and are less suppressive than their control counterparts *in vitro* (157). Increased frequencies of Th1-like T_regs_ have been found in patients with MS, type 1 diabetes, and in mouse models of Sjögren Syndrome, and are thought to contribute to the loss of tolerance in autoimmune diseases (158). Th1-like T_regs_ secrete IFN-γ *via* activation of the PI3K-AKT-FOXO1/3 pathway, and using an *in vitro* approach, activation of PI3K has been shown to be sufficient to induce a Th1-like phenotype in human T_regs_ (159, 160). Given that mTORC1 is downstream of the PI3K pathway it is possible that alterations in cellular metabolism also accompany such examples of T_reg_ plasticity.

Development of MS is thought to arise from unfavorable interactions between genetic risk loci and environmental conditions and/or cues. Numerous environmental triggers have been proposed, including smoking, vitamin D, EBV infection, salt, and gut microbiota (161, 162). However, none of these factors reconcile the rise in MS incidence in the western world, except the changes in diet and consequential rise in obesity. The Western diet contains greater concentrations of salt, refined sugars, and unsaturated fats (163). We have reported that the physiologic sodium concentrations induce an inflammatory signature in T_eff_ cells and drives a Th1-like phenotype in T_regs_ (2). Further, obesity has been identified as another risk factor for the development of MS, and adipose tissue of obese patients has been shown to host a chronic, pro-inflammatory environment, indicating that diet can influence immune cell activation and phenotypes within tissues, which might play an important role in MS (164, 165).

### Altered Lipid Profiles in Multiple Sclerosis

Lipids play many roles in the CNS including signaling, structural support, mediating inflammation, and membrane biogenesis (9). Therefore, during inflammation changes in the availability and profiles of FFA species might alter disease pathogenesis by influencing immune cell function. In multiple sclerosis, immune cells attack the myelin sheath around nerves that are rich in lipids, containing approximately 700 different lipid species (11, 15, 16), and as a result many studies have found altered lipid profiles in the MS patients and EAE models (8, 166), that could potentially serve as biomarkers of the disease. At the time of diagnosis, the CSF of MS patients harbor differences in many lipid species, including pro-inflammatory arachidonic acid, compared to the non-MS control group (167). In support of these data, lipid profiling of mouse EAE brains showed that during acute inflammation, lipid metabolism is shifted from the pathway that creates common lipid substrates into the pathway producing the pro-inflammatory arachidonic acid (168). Studies profiling the phospholipids of serum samples from patients with MS have also demonstrated that patients with MS have a phospholipidomic signature different from that of healthy controls (169).

### Altered T Cell Metabolic Profiles in Multiple Sclerosis

As with infections and metabolic syndromes, the inflammation induced in autoimmunity triggers metabolic alterations locally and systemically (166). In the blood of patients with MS and other autoimmune disorders such as rheumatoid arthritis and systemic lupus erythematosus, there are elevated levels of IL-1β, IL-6, TGF-β, and IL-23, which promote pathogenic Th17 cell differentiation and block T_reg_ development *in vivo* and *in vitro* (170–176), proving that there is a disturbance in the balance between the induction and regulation of pro-inflammatory T_eff_ cells. Although the metabolic cues that shift this balance are still being defined, there is evidence that glycolysis and OXPHOS are impaired during T cell activation in relapsing-remitting MS patients (177).

**Figure 3:**
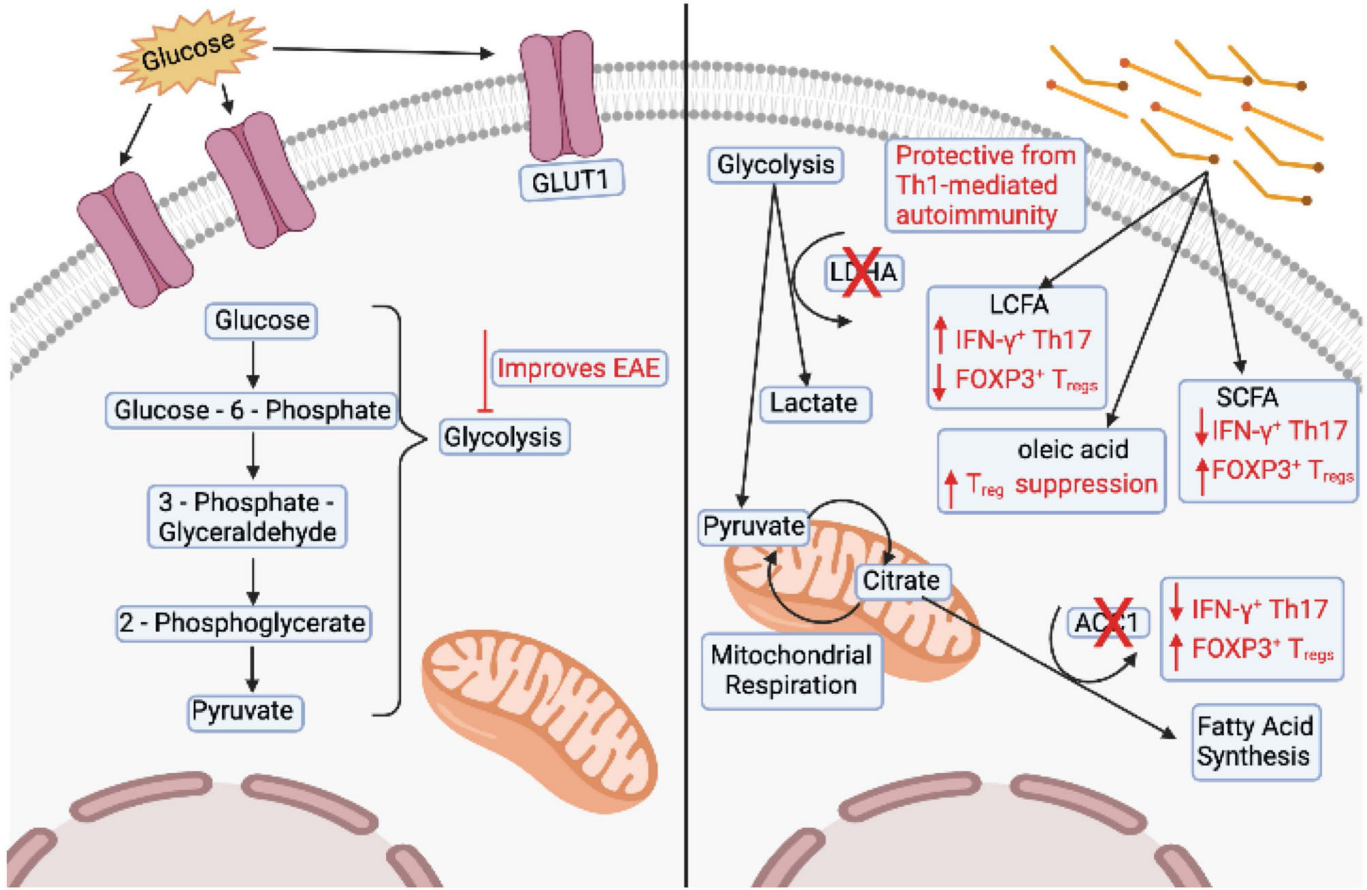
Changes in T cells associated with multiple sclerosis. The left panel shows overexpression of GLUT1 and dysregulation of glycolysis associated with T cells in experimental autoimmune encephalomyelitis (EAE) models. Inhibition of glycolysis also improves clinical outcomes of EAE. The right panel shows that deletion of lactate dehydrogenase (LDHA) is protective in Th1-mediated autoimmunity. Deletion of acetyl-CoA carboxylase 1 (ACC1) in T cells decreases the number of interferon (IFN)-γ+ Th17 cells and increases the number of FOXP3+ Treg cells. Mice fed long chain fatty acids (LCFAs) have an increased number of IFN-γ+ and IL-17+ T cells and a decreased number of FOXP3+ cells. Conversely, mice fed short chain fatty acids (SCFAs) have an decreased number of IFN-γ+ and IL-17+ T cells and an increased number of FOXP3+ cells. Finally, T cells cultured in the presence of LCFA, oleic acid, can partially restore Treg suppression. Image created with BioRender.com

As mentioned above, glycolysis is a common feature of Th1 and Th17 CD4^+^ T_eff_ cells (13, 47). Inhibiting glucose metabolism can inhibit differentiation of Th1 and Th17 lineages even in the presence of polarizing cytokine (31, 47). Further, blocking glycolysis using 2-deoxyglucose (2-DG) improves clinical outcomes in EAE models (47) (**Figure 3**). A direct link between dysregulated glucose metabolism in lymphocytes and autoimmunity was shown in mice that over-express GLUT1 in T cells (178). Increased glucose uptake drives IFN-γ and IL-2 expression in T cells upon *in vitro* stimulation, and deletion of *GLUT1* reduces glycolysis in CD4^+^ T cells, and effector differentiation. These cells are also unable to provide protection in a model of colitis (34). Further, CD4^+^ T cells in lupus-prone mice show increases in glycolytic engagement compared to healthy control cells (179).

Some studies have reported alterations in T cell lipid metabolism as a contributor to MS pathogenesis (180–183). Activated T cells have higher cholesterol and fatty acid concentrations in their membrane (184, 185), therefore altered lipid-mediated signaling could contribute to MS pathogenesis (186). Specific deletion of ACC1 in T cells protects mice from EAE by reducing the number of IFN-γ^+^ Th17 cells and increases the number of FOXP3^+^ T_regs_ in the spinal cord (42). Conditional deletion of LDHA in T cells protects susceptible mice from Th1-mediated autoimmunity (35). Simvastatin, an inhibitor of cholesterol biosynthesis, was used in a clinical trial to treat MS and was able to attenuate brain atrophy and disease progression (187). In support, during the chronic phase of EAE, linoleic and cholesterol metabolism have been found to be altered (9, 10), and inhibition of cholesterol synthesis *via* statins, prevented EAE progression by blocking Th17 differentiation (188). However, statins broadly affect the immune system, including promotion of Th2 cells and inhibition of the cytokines that induce Th1 and Th17 differentiation, and therefore their effect on MS might not be directly related to their effect on cholesterol (187–189).

Long chain fatty acids (LCFAs) and short chain fatty acids (SCFAs) have been shown to exert opposing effects on T cell differentiation. LCFAs promote IFN-γ and IL-17 production and exacerbate EAE, whereas SCFAs induce FOXP3 expression and provide protection (5). We have recently shown that T_regs_ isolated from the adipose tissue of MS patients exhibit a pro-inflammatory transcriptome that resembles the transcriptome of T_regs_ treated *in vitro* with the pro-inflammatory LCFA arachidonic acid (4). T_regs_ isolated from the adipose of healthy subjects and T_regs_ exposed to oleic acid *in vitro* do not show an inflammatory transcriptome, but rather exhibit increased FAO-driven OXPHOS metabolism, creating a positive feedback mechanism that induces the expression of FOXP3 and enhances phosphorylation of STAT5, which acts to stabilize the T_reg_ lineage and increase suppressive function (4). Finally, treating dysfunctional MS T_regs_ with oleic acid can partially restore their suppressive function, highlighting a role for dietary lipids in shaping T_reg_ function and identity in MS (4, 92, 190, 191) (**Figure 3**). Further studies must address how metabolic perturbations generate and sustain pathogenic T cells in the context of MS and other autoimmune diseases. Metabolic signatures of T cells are intricately linked to their differentiation and activation status. However, under inflammatory conditions such as infection or during metabolic syndromes, autoimmunity, and cancer, the microenvironment drastically changes in regard to nutritional availability, forcing T cells to adapt to these changing environments.

Future studies focusing on how nutrient depletion or metabolite availability shapes metabolic utilization and differentiation of T_eff_ and T_reg_ cells will be critical to our understanding and implementation of targeted therapies. For example, Shi et al. have shown that blocking glycolysis improves clinical outcomes of EAE (47), and disease-modulatory effects of feeding mice both LCFAs and SCFAs have been observed (5). However, to realistically translate these data to human therapies we must first develop more reliable metabolic profiles of T cells and other cells involved in disease pathogenesis and progression during active disease states. Once established, we can couple these data to cytokine profiles or other functional readouts, e.g. suppressive capacity of T_regs_, in order to more precisely define relevant metabolic targets. One current limitation of metabolic therapies is that the target molecules are often the regulators of central metabolic nodes that integrate many signals and crosstalk between multiple pathways, such as mTOR. However, the ability to fine tune T cell functions and phenotypes that are more favorable for disease outcomes will depend on specifically targeting certain metabolic intermediates.

## Conclusions

Lipids are a critical component of all cellular and organelle membranes. They provide structural support to the cell, serve as fuel, and signaling platforms. Lipid species are as diverse as proteins, however, limitations in technologies to study lipids have left major gaps in our understanding of their range of impacting biological functions, especially in lymphocytes. Despite these gaps, we know that different T cell subsets differentially utilize lipids for fuel and function (192). Upon activation of an T_eff_ cell, there is a switch from a predominantly OXPHOS metabolic program to aerobic glycolysis, a more anabolic process critical for growth and proliferation during an immune response (47, 192). Conversely, OXPHOS mainly supports survival of T_regs_ and memory T cells (31). Although these cell types rely on the same metabolic program, they acquire and utilize lipid species differently (15). Given the diversity of FFAs, profiling lipid composition in tissues is crucial to understand which FFA species might be important in regulating T cell function in specific microenvironments and physiological states. Current data show the pleiotropic effects lipid species exert on T cells, depending on fatty acid length, degree of saturation, and nutrient availability in the environment. There are clear correlations and fluctuations in lipid profiles with diet and disease states, so understanding these differences will be critical to discern how lipid signaling and lipid-driven metabolism affects the function and phenotypes of T cells in disease states. There are many remaining questions regarding fatty acid-specific effects on T cell function. For example, it is not well defined which FFA are preferentially metabolized, which can serve as signaling molecules, or which FFA exert their effects by increasing intermediates generated by FAO or other downstream metabolic processes. It is also not fully understood if lipid receptors display degrees of specificity beyond FFA length. Better characterization of these receptor families could potentially provide insight as to which FFA are being utilized by T cell subsets and how the receptor expression profile might shift in specific disease states. Regardless, given the constant exposure of T cells to lipid species in the tissues, it is certain these molecules have important implications in T cell biology.

## Author Contributions

SLP wrote the manuscript under the supervision of MD-V and DAH. Correspondence should be addressed to MD-V. All authors contributed to the article and approved the submitted version.

## Funding

This work was supported by grants to DAH from the NIH (U19 AI089992, R25 NS079193, P01 AI073748, U24 AI11867, R01 AI22220, UM 1HG009390, P01 AI039671, P50 CA121974, and R01 CA227473); the National Multiple Sclerosis Society (NMSS) (CA 1061- A-18, RG-1802-30153); the Nancy Taylor Foundation for Chronic Diseases; and Race to Erase MS.

## Conflict of Interest

DH has received funding for his laboratory from Bristol Myers Squibb and Genentech. A child of DAH is an employee of Sanofi. Further information regarding funding is available at: https://openpaymentsdata.cms.gov/physician/166753.

The remaining authors declare that the research was conducted in the absence of any commercial or financial relationships that could be construed as a potential conflict of interest.

## Publisher’s Note

All claims expressed in this article are solely those of the authors and do not necessarily represent those of their affiliated organizations, or those of the publisher, the editors and the reviewers. Any product that may be evaluated in this article, or claim that may be made by its manufacturer, is not guaranteed or endorsed by the publisher.

## Abbreviations

ACC1, Acetyl-CoA carboxylase-1; ATP, Adenosine triphosphate; AMPK, AMP activated protein kinase; CNS, Central nervous system; CSF, Cerebrospinal fluid; DHA, Docosahexaenoic acid; T_eff_, Effector T cells; EPA, Eicosapentaenoic acid; ETC, Electron transport chain; EAE, Experimental autoimmune encephalomyelitis; FAO, Fatty acid oxidation; T_fh_, Follicular T helper cells; FFA, Free fatty acid; GM-CSF, Granulocyte macrophage colony-stimulating factor; LCFA, Long chain fatty acids; IFN-γ, Interferon gamma; IL, Interleukin; LDHA, Lactate dehydrogenase; mTOR, Mammalian target of rapamycin; MCFA, Medium chain fatty acids; MS, Multiple sclerosis; OXPHOS, Oxidative phosphorylation; pT_reg_, Peripheral T_reg_; PPAR, Peroxisome proliferator-activated receptor; PTEN, Phosphatase and tensin homolog; PI3K, Phosphatidylinositol 3-kinase; PDK1, Pyruvate dehydrogenase kinase 1; Rheb, Ras homologue enriched in brain; Rictor, Rapamycin-insensitive companion of TOR; T_reg_, Regulatory T cells; SCFA, Short chain fatty acids; TCR, T cell receptor; Th, T helper; tT_reg_, Thymic T_reg_; TGF, Transforming growth factor; TCA, Tricarboxylic acid.

## References

[1] Klocperk A Grecová J Šišmová K Kayserová J Froňková E Šedivá A. Helios Expression in T-Regulatory Cells in Patients With Di George Syndrome. J Clin Immunol (2014) 34:864–70. doi: 10.1007/s10875-014-0071-y 25008482

[2] Hernandez AL Kitz A Wu C Lowther DE Rodriguez DM Vudattu N. Sodiu Chlorde Inhibits the Suppressive Function of FOXP3+ Regulatory T Cells. J Clin Invest (2015) 125:4212–22. doi: 10.1172/JCI81151 PMC4639983 26524592

[3] Kleinewietfeld M Manzel A Titze J Kvakan H Yosef N Linker R. Sodium Chloride Drives Autoimmune Disease by the Induction of Pathogenic T(H)17 Cells. Nature (2013) 496:518. doi: 10.1038/nature11868 23467095 PMC3746493

[4] Pompura SL Wagner A Kitz A LaPerche J Yosef N Dominguez-Villar M. Oleic Acid Restores Suppressive Defects in Tissue-Resident FOXP3 Tregs From Patients With Multiple Sclerosis. J Clin Invest (2021) 131(2):e138519 doi: 10.1172/JCI138519 PMC7810477 33170805

[5] Haghikia A Jörg S Duscha A Berg J Manzel A Waschbisch A. Dietary Fatty Acids Directly Impact Central Nervous System Autoimmunity *via* the Small Intestine. Immunity (2015) 43:817–29. doi: 10.1016/j.immuni.2015.09.007 26488817

[6] Mendoza A Fang V Chen C Serasinghe M Verma A Mulle J. Lymphatic Endothelial S1P Promotes Mitochondrial Function and Survival in Naive T Cells. Nature (2017) 546:158. doi: 10.1038/nature22352 28538737 PMC5683179

[7] Endo Y Asou HK Matsugae N Hirahara K Shinoda K Tumes DJ. Obesity Drives Th17 Cell Differentiation by Inducing the Lipid Metabolic Kinase, Acc1. Cell Rep (2015) 12:1042–55. doi: 10.1016/j.celrep.2015.07.014 26235623

[8] Bhargava P Calabresi PA. Metabolomics in Multiple Sclerosis. Multiple Sclerosis J (2016) 22:451–60. doi: 10.1177/1352458515622827 26754801

[9] Giri S Laila P Singh J Suhail H Deshpande M Datta I. Profile of Circulatory Metabolites in Chronic Mouse Model of Multiple Sclerosis Using Untargeted Global Metabolomics. J Immunol (2014) 192:10.

[10] Poisson LM Suhail H Singh J Datta I Denic A Labuzek K. Untargeted Plasma Metabolomics Identifies Endogenous Metabolite With Drug-Like Properties in Chronic Animal Model of Multiple Sclerosis. J Biol Chem (2015) 290(52):30697–712. doi: 10.1074/jbc.M115.679068 PMC4692201 26546682

[11] Warburg O Wind F Negelein E. The Metabolism of Tumors in the Body. J Gen Physiol (1927) 8:519–30. doi: 10.1085/jgp.8.6.519 PMC2140820 19872213

[12] Heiden MGV Cantley LC Thompson CB. Understanding the Warburg Effect: The Metabolic Requirements of Cell Proliferation. Science (2009) 324:1029–33. doi: 10.1126/science.1160809 PMC2849637 19460998

[13] Wang R Dillon C Shi LZ Milasta S Carter R Finkelstein D. The Transcription Factor Myc Controls Metabolic Reprogramming Upon T Lymphocyte Activation. Immunity (2011) 35:871–82. doi: 10.1016/j.immuni.2011.09.021 PMC3248798 22195744

[14] Sena LA Li S Jairaman A Prakriya M Ezponda T Hildeman DA. Mitochondria Are Required for Antigen-Specific T Cell Activation Through Reactive Oxygen Species Signaling. Immunity (2013) 38:225–36. doi: 10.1016/j.immuni.2012.10.020 PMC3582741 23415911

[15] Pollizzi KN Patel CH Sun IH Oh MH Waickman AT Wen J. Mtorc1 and Mtorc2 Selectively Regulate CD8(+) T Cell Differentiation. J Clin Invest (2015) 125:2090–108. doi: 10.1172/JCI77746 PMC4463194 25893604

[16] Yang K Neale G Green DR He W Chi H. The Tumor Suppressor Tsc1 Enforces Quiescence of Naive T Cells to Promote Immune Homeostasis and Function. Nat Immunol (2011) 12:888–U811. doi: 10.1038/ni.2068 21765414 PMC3158818

[17] Saxton RA Sabatini DM. mTOR Signaling in Growth, Metabolism, and Disease. Cell (2017) 168:960–76. doi: 10.1016/j.cell.2017.02.004 PMC5394987 28283069

[18] Inoki K Zhu TQ Guan KL. TSC2 Mediates Cellular Energy Response to Control Cell Growth and Survival. Cell (2003) 115:577–90. doi: 10.1016/S0092-8674(03)00929-2 14651849

[19] Frauwirth KA Riley JL Harris MH Parry RV Rathmell JC Plas DR. The CD28 Signaling Pathway Regulates Glucose Metabolism. Immunity (2002) 16:769–77. doi: 10.1016/S1074-7613(02)00323-0 12121659

[20] MacIver NJ Blagih J Saucillo DC Tonelli L Griss T Rathmell JC. The Liver Kinase B1 Is a Central Regulator of T Cell Development, Activation, and Metabolism. J Immunol (2011) 187:4187–98. doi: 10.4049/jimmunol.1100367 PMC3206094 21930968

[21] Tamás P Hawley SA Clarke RG Mustard KJ Green K Hardie DG. Regulation of the Energy Sensor AMP-Activated Protein Kinase by Antigen Receptor and Ca2+ in T Lymphocytes. J Exp Med (2006) 203:1665–70. doi: 10.1084/jem.20052469 PMC2118355 16818670

[22] Blagih J Coulombe F Vincent EE Dupuy F Vázquez GC Yurchenko Ekaterina. The Energy Sensor AMPK Regulates T Cell Metabolic Adaptation and Effector Responses *In Vivo* . Immunity (2015) 42:41–54. doi: 10.1016/j.immuni.2014.12.030 25607458

[23] Adams WC Chen YH Kratchmarov R Yen B Nish SA Lin WHW. Anabolism-Associated Mitochondrial Stasis Driving Lymphocyte Differentiation Over Self-Renewal. Cell Rep (2016) 17:3142–52. doi: 10.1016/j.celrep.2016.11.065 PMC5189677 28009285

[24] Kinnaird A Zhao S Wellen KE Michelakis ED. Metabolic Control of Epigenetics in Cancer. Nat Rev Cancer (2016) 16:694–707. doi: 10.1038/nrc.2016.82 27634449

[25] Chang C-H Curtis JD Maggi LB Jr Faubert B Villarino AV O’Sullivan D. Posttranscriptional Control of T Cell Effector Function by Aerobic Glycolysis. Cell (2013) 153:1239–51. doi: 10.1016/j.cell.2013.05.016 PMC3804311 23746840

[26] Tarasenko TN Pacheco SE Koenig MK Gomez-Rodriguez J Kapnick SM Diaz F. Cytochrome C Oxidase Activity Is a Metabolic Checkpoint That Regulates Cell Fate Decisions During T Cell Activation and Differentiation. Cell Metab (2017) 25:1254. doi: 10.1016/j.cmet.2017.05.007 28591633 PMC5562283

[27] Jackson SH Devadas S Kwon J Pinto LA Williams MS. T Cells Express a Phagocyte-Type NADPH Oxidase That Is Activated After T Cell Receptor Stimulation. Nat Immunol (2004) 5:818–27. doi: 10.1038/ni1096 15258578

[28] Hosios AM Hecht VC Danai LV Johnson MO Rathmell JC Steinhauser ML. Amino Acids Rather Than Glucose Account for the Majority of Cell Mass in Proliferating Mammalian Cells. Dev Cell (2016) 36:540–9. doi: 10.1016/j.devcel.2016.02.012 PMC4766004 26954548

[29] Liberti MV Locasale JW. The Warburg Effect: How Does it Benefit Cancer Cells? Trends Biochem Sci (2016) 41:211–8. doi: 10.1016/j.tibs.2015.12.001 PMC4783224 26778478

[30] Fox CJ Hammerman PS Thompson CB. Fuel Feeds Function: Energy Metabolism and the T-Cell Response. Nat Rev Immunol (2005) 5:844–52. doi: 10.1038/nri1710 16239903

[31] Michalek RD Gerriets VA Jacobs SR Macintyre AN MacIver NJ Mason EF. Cutting Edge: Distinct Glycolytic and Lipid Oxidative Metabolic Programs Are Essential for Effector and Regulatory CD4(+) T Cell Subsets. J Immunol (2011) 186:3299–303. doi: 10.4049/jimmunol.1003613 PMC3198034 21317389

[32] Klysz D Tai X Robert PA Craveiro M Cretenet G Oburoglu L. Glutamine-Dependent Alpha-Ketoglutarate Production Regulates the Balance Between T Helper 1 Cell and Regulatory T Cell Generation. Sci Signaling (2015) 8(396):ra97. doi: 10.1126/scisignal.aab2610 26420908

[33] Chornoguz O Hagan RS Haile A Arwood ML Gamper CJ Banerjee A. Mtorc1 Promotes T-Bet Phosphorylation To Regulate Th1 Differentiation. J Immunol (2017) 198:3939–48. doi: 10.4049/jimmunol.1601078 PMC5458608 28424242

[34] Macintyre AN Gerriets VA Nichols AG Michalek RD Rudolph MC Deoliveira D. The Glucose Transporter Glut1 Is Selectively Essential for CD4 T Cell Activation and Effector Function. Cell Metab (2014) 20:61–72. doi: 10.1016/j.cmet.2014.05.004 24930970 PMC4079750

[35] Peng M Yin N Chhangawala S Xu K Leslie CS Li MO. Aerobic Glycolysis Promotes T Helper 1 Cell Differentiation Through an Epigenetic Mechanism. Science (2016) 354:481–4. doi: 10.1126/science.aaf6284 PMC5539971 27708054

[36] Cham CM Driessens G O’Keefe JP Gajewski TF. Glucose Deprivation Inhibits Multiple Key Gene Expression Events and Effector Functions in CD8(+) T Cells. Eur J Immunol (2008) 38:2438–50. doi: 10.1002/eji.200838289 PMC3008428 18792400

[37] Cham CM Gajewski TF. Glucose Availability Regulates IFN-Gamma Production and P70s6 Kinase Activation in CD8(+) Effector T Cells. J Immunol (2005) 174:4670–7. doi: 10.4049/jimmunol.174.8.4670 15814691

[38] Gubser PM Bantug GR Razik L Fischer M Dimeloe S Hoenger G. Rapid Effector Function of Memory CD8(+) T Cells Requires an Immediate-Early Glycolytic Switch. Nat Immunol (2013) 14:1064. doi: 10.1038/ni.2687 23955661

[39] Bailis W Shyer JA Zhao J Canaveras JCG Al Khazal FJ Qu R. Distinct Modes of Mitochondrial Metabolism Uncouple T Cell Differentiation and Function. Nature (2019) 571:403. doi: 10.1038/s41586-019-1311-3 31217581 PMC6939459

[40] Franchi L Monteleone I Hao L-Y Spahr MA Zhao W Liu X. Inhibiting Oxidative Phosphorylation *In Vivo* Restrains Th17 Effector Responses and Ameliorates Murine Colitis. J Immunol (2017) 198:2735–46. doi: 10.4049/jimmunol.1600810 PMC5360504 28242647

[41] Gerriets VA Kishton RJ Nichols AG Macintyre AN Inoue M Ilkayeva O. Metabolic Programming and PDHK1 Control CD4(+) T Cell Subsets and Inflammation. J Clin Invest (2015) 125:194–207. doi: 10.1172/JCI76012 25437876 PMC4382238

[42] Berod L Friedrich C Nandan A Freitag J Hagemann S Harmrolfs K. *De Novo* Fatty Acid Synthesis Controls the Fate Between Regulatory T and T Helper 17 Cells. Nat Med (2014) 20:1327–33. doi: 10.1038/nm.3704 25282359

[43] Gaublomme JT Yosef N Lee Y Gertner RS Yang LV Wu C. Single-Cell Genomics Unveils Critical Regulators of Th17 Cell Pathogenicity. Cell (2015) 163(6):1400–12. doi: 10.1016/j.cell.2015.11.009 PMC4671824 26607794

[44] Hu X Wang Y Hao L-Y Liu X Lesch CA Sanchez BM. Sterol Metabolism Controls T(H)17 Differentiation by Generating Endogenous ROR Gamma Agonists. Nat Chem Biol (2015) 11(2):141–U184. doi: 10.1038/nchembio.1714 25558972

[45] Santori FR Huang P van dePavert SA Douglass EF Jr Leaver DJ Haubrich BA. Identification of Natural ROR Gamma Ligands That Regulate the Development of Lymphoid Cells. Cell Metab (2015) 21:286–97. doi: 10.1016/j.cmet.2015.01.004 PMC4317570 25651181

[46] Wang C Yosef N Gaublomme J Wu C Lee Y Clish CB. CD5L/AIM Regulates Lipid Biosynthesis and Restrains Th17 Cell Pathogenicity. Cell (2015) 163(6):1413–27. doi: 10.1016/j.cell.2015.10.068 PMC4671820 26607793

[47] Shi LZ Wang R Huang G Vogel P Neale G Green DR. HIF1 Alpha-Dependent Glycolytic Pathway Orchestrates a Metabolic Checkpoint for the Differentiation of T(H)17 and T-Reg Cells. J Exp Med (2011) 208:1367–76. doi: 10.1084/jem.20110278 PMC3135370 21708926

[48] Delgoffe GM Kole TP Zheng Y Zarek PE Matthews KL Xiao B. The mTOR Kinase Differentially Regulates Effector and Regulatory T Cell Lineage Commitment. Immunity (2009) 30:832–44. doi: 10.1016/j.immuni.2009.04.014 PMC2768135 19538929

[49] Heikamp EB Patel CH Collins S Waickman A Oh M-H Sun I-H. The AGC Kinase SGK1 Regulates T(H)1 and T(H)2 Differentiation Downstream of the Mtorc2 Complex. Nat Immunol (2014) 15:457. doi: 10.1038/ni.2867 24705297 PMC4267697

[50] Yang J-Q Kalim KW Li Y Zhang S Hinge A Filippi M-D. RhoA Orchestrates Glycolysis for T(H)2 Cell Differentiation and Allergic Airway Inflammation. J Allergy Clin Immunol (2016) 137:231. doi: 10.1016/j.jaci.2015.05.004 26100081 PMC4684821

[51] Seumois G Zapardiel-Gonzalo J White B Singh D Schulten V Dillon M. Transcriptional Profiling of Th2 Cells Identifies Pathogenic Features Associated With Asthma. J Immunol (2016) 197:655–64. doi: 10.4049/jimmunol.1600397 PMC4936908 27271570

[52] Chen T Tibbitt CA Feng X Stark JM Rohrbeck L Rausch L. PPAR-Gamma Promotes Type 2 Immune Responses in Allergy and Nematode Infection. Sci Immunol (2017) 2(9):eaal5196. doi: 10.1126/sciimmunol.aal5196 28783701

[53] Procaccini C Carbone F Di Silvestre D Brambilla F De Rosa V Galgani M. The Proteomic Landscape of Human Ex Vivo Regulatory and Conventional T Cells Reveals Specific Metabolic Requirements. Immunity (2016) 44:712–2. doi: 10.1016/j.immuni.2016.02.022 PMC5641922 28843073

[54] Beura LK Hamilton SE Bi K Schenkel JM Odumade OA Casey KA. Normalizing the Environment Recapitulates Adult Human Immune Traits in Laboratory Mice. Nature (2016) 532:512. doi: 10.1038/nature17655 27096360 PMC4871315

[55] Chapman NM Zeng H Nguyen T-LM Wang Y Vogel P Dhungana Y. mTOR Coordinates Transcriptional Programs and Mitochondrial Metabolism of Activated T-Reg Subsets to Protect Tissue Homeostasis. Nat Commun (2018) 9(1):2095. doi: 10.1038/s41467-018-04392-5 29844370 PMC5974344

[56] Asanuma S Tanaka J Sugita J Kosugi M Shiratori S Wakasa K. Expansion of CD4(+)CD25(+) Regulatory T Cells From Cord Blood CD4(+) Cells Using the Common Gamma-Chain Cytokines (IL-2 and IL-15) and Rapamycin. Ann Hematol (2011) 90:. doi: 10.1007/s00277-010-1121-z 21107839

[57] Raud B Roy DG Divakaruni AS Tarasenko TN Franke R Ma EH. Etomoxir Actions on Regulatory and Memory T Cells Are Independent of Cpt1a-Mediated Fatty Acid Oxidation. Cell Metab (2018) 28:504. doi: 10.1016/j.cmet.2018.06.002 30043753 PMC6747686

[58] Sun Y Tian T Gao J Liu X Hou H Cao R. Metformin Ameliorates the Development of Experimental Autoimmune Encephalomyelitis by Regulating T Helper 17 and Regulatory T Cells in Mice. J Neuroimmunol (2016) 292:58–67. doi: 10.1016/j.jneuroim.2016.01.014 26943960

[59] Lee S-Y Lee SH Yang E-J Kim E-K Kim J-K Shin D-Y. Metformin Ameliorates Inflammatory Bowel Disease by Suppression of the STAT3 Signaling Pathway and Regulation of the Between Th17/Treg Balance. PloS One (2015) 10(9):e0135858. doi: 10.1371/journal.pone.0135858 26360050 PMC4567351

[60] Gerriets VA. Foxp3 and Toll-Like Receptor Signaling Balance T-Reg Cell Anabolic Metabolism for Suppression. Nat Immunol (2016) 17:1459–66. doi: 10.1038/ni.3577 PMC5215903 27695003

[61] Kopf H de la Rosa GM Howard OMZ Chen X. Rapamycin Inhibits Differentiation of Th17 Cells and Promotes Generation of Foxp3+T Regulatory Cells. Int Immunopharmacol (2007) 7:1819–24. doi: 10.1016/j.intimp.2007.08.027 PMC2223142 17996694

[62] Apostolidis SA Rodríguez-Rodríguez N Suárez-Fueyo A Dioufa N Ozcan E Crispín JC. Phosphatase PP2A is Requisite for the Function of Regulatory T Cells. Nat Immunol (2016) 17:556. doi: 10.1038/ni.3390 26974206 PMC4837024

[63] Basu S Hubbard B Shevach EM. Foxp3-Mediated Inhibition of Akt Inhibits Glut1 (Glucose Transporter 1) Expression in Human T Regulatory Cells. J Leukocyte Biol (2015) 97:279–83. doi: 10.1189/jlb.2AB0514-273RR PMC4304425 25492937

[64] Angelin A Gil-de-Gómez L Dahiya S Jiao J Guo L Levine MH. Foxp3 Reprograms T Cell Metabolism to Function in Low-Glucose, High-Lactate Environments. Cell Metab (2017) 25:1282. doi: 10.1016/j.cmet.2016.12.018 28416194 PMC5462872

[65] Kishore M Cheung KCP Fu H Bonacina F Wang G Coe D. Regulatory T Cell Migration Is Dependent on Glucokinase-Mediated Glycolysis. Immunity (2017) 47(5):875–889.e10. doi: 10.1016/j.immuni.2017.10.017 29166588 PMC5714502

[66] Travis MA Sheppard D. TGF-β Activation and Function in Immunity Annu Rev Immunol (2014). 32:51-82. doi: 10.1146/annurev-immunol-032713-120257 24313777 PMC4010192

[67] Xie M Zhang D Dyck JRB Li Y Zhang H Morishima M. A Pivotal Role for Endogenous TGF-Beta-Activated Kinase-1 in the LKB1/AMP-Activated Protein Kinase Energy-Sensor Pathway. Proc Natl Acad Sci USA (2006) 103:17378–83. doi: 10.1073/pnas.0604708103 PMC1859937 17085580

[68] Gualdoni GA Mayer K Göschl L Boucheron N Ellmeier W Zlabinge GJ. The AMP Analog AICAR Modulates the T-Reg/T(h)17 Axis Through Enhancement of Fatty Acid Oxidation. FASEB J (2016) 30:3800–9. doi: 10.1096/fj.201600522R 27492924

[69] Huynh A DuPage M Priyadharshini B Sage PT Quiros J Borges CM. Control of PI(3) Kinase in T-Reg Cells Maintains Homeostasis and Lineage Stability. Nat Immunol (2015) 16:188. doi: 10.1038/ni.3077 25559257 PMC4297515

[70] Sharmahul MD Shinde R Mcgaha L Huang L Holmgaard RB Wolchok JD. The PTEN Pathway in T-Regs is a Critical Driver of the Suppressive Tumor Microenvironment. Sci Adv (2015) 1(10):e1500845. doi: 10.1126/sciadv.1500845 26601142 PMC4640592

[71] Weinberg SE Singer BD Steinert EM Martinez CA Mehta MM Martínez-Reyes I. Mitochondrial Complex III is Essential for Suppressive Function of Regulatory T Cells. Nature (2019) 565:495. doi: 10.1038/s41586-018-0846-z 30626970 PMC6345596

[72] De Rosa V Galgani M Porcellini A Colamatteo A Santopaolo M Zuchegna C. Glycolysis Controls the Induction of Human Regulatory T Cells by Modulating the Expression of FOXP3 Exon 2 Splicing Variants. Nat Immunol (2015) 16:1174–84. doi: 10.1038/ni.3269 PMC4868085 26414764

[73] Zeng H Yang K Cloer C Neale G Vogel P Chi H. Mtorc1 Couples Immune Signals and Metabolic Programming to Establish T-Reg-Cell Function. Nature (2013) 499:485. doi: 10.1038/nature12297 23812589 PMC3759242

[74] Procaccini C De Rosa V Galgani M Abanni L Calì G Porcellini A. An Oscillatory Switch in mTOR Kinase Activity Sets Regulatory T Cell Responsiveness. Immunity (2010) 33:929–41. doi: 10.1016/j.immuni.2010.11.024 PMC3133602 21145759

[75] Rueda CM Rodríguez-Perea AL Moreno-Fernandez M Jackson CM Melchior JT Davidson WS. High Density Lipoproteins Selectively Promote the Survival of Human Regulatory T Cells. J Lipid Res (2017) 58:1514–23. doi: 10.1194/jlr.M072835 PMC5538275 28377425

[76] de Jong AJ Kloppenburg M Toes REM Ioan-Facsinay A. Fatty Acids, Lipid Mediators, and T-Cell Function. Front Immunol (2014) 5. doi: 10.3389/fimmu.2014.00483 PMC4195378 25352844

[77] Cury-Boaventura MF Gorjao R de Lima TM Newsholme P Curi R. Comparative Toxicity of Oleic and Linoleic Acid on Human Lymphocytes (Vol 78, Pg 1448, 2006). Life Sci (2014) 112:97–7. doi: 10.1016/j.lfs.2013.01.014 16236329

[78] Takahashi HK Cambiaghi TD Luchessi AD Hirabara SM Vinolo MAR Newsholme P. Activation of Survival and Apoptotic Signaling Pathways in Lymphocytes Exposed to Palmitic Acid. J Cell Physiol (2012) 227(1):339–50. doi: 10.1002/jcp.22740 21437903

[79] Howie D Cobbold SP Adams E Bokum AT Necula AS Zhang W. Foxp3 Drives Oxidative Phosphorylation and Protection From Lipotoxicity. JCI Insight (2017) 2(3):e89160. doi: 10.1172/jci.insight.89160 28194435 PMC5291728

[80] Xu S Nam SM Kim J-H Das R Choi S-K Nguyen TT. Palmitate Induces ER Calcium Depletion and Apoptosis in Mouse Podocytes Subsequent to Mitochondrial Oxidative Stress. Cell Death Dis (2015) 6(11):e1976. doi: 10.1038/cddis.2015.331 26583319 PMC4670935

[81] Spector AA. Plasma Lipid Transport. Clin Physiol Biochem (1984) 2(2–3):123–34. 6386279

[82] Calder PC. Omega-3 Fatty Acids and Inflammatory Processes. Nutrients (2010) 2(3):355–74. doi: 10.3390/nu2030355 PMC3257651 22254027

[83] Innes JK Calder PC. Omega-6 Fatty Acids and Inflammation. Prostaglandins Leukotrienes Essential Fatty Acids (2018) 132:41–8. doi: 10.1016/j.plefa.2018.03.004 29610056

[84] Field CJ Clandinin MT. Modulation Of Adipose-Tissue Fat Composition By Diet - A Review. Nutr Res (1984) 4:743–55. doi: 10.1016/S0271-5317(84)80050-0

[85] Body DR. The Lipid-Composition Of Adipose-Tissue. Prog Lipid Res (1988) 27:39–60. doi: 10.1016/0163-7827(88)90004-5 3057509

[86] Corsetto PA Montorfano G Zava S Jovenitti IE Cremona A Berra B. Effects of N-3 PUFAs on Breast Cancer Cells Through Their Incorporation in Plasma Membrane. Lipids Health Dis (2011) 10:73. doi: 10.1186/1476-511X-10-73 21569413 PMC3127786

[87] Kambe T Murakami M Kudo I. Polyunsaturated Fatty Acids Potentiate Interleukin-1-Stimulated Arachidonic Acid Release by Cells Overexpressing Type IIA Secretory Phospholipase a(2). FEBS Lett (1999) 453:81–4. doi: 10.1016/S0014-5793(99)00702-4 10403380

[88] Kolodin D van Panhuys N Li C Magnuson AM Cipolletta D Miller C M. Antigen- and Cytokine-Driven Accumulation of Regulatory T Cells in Visceral Adipose Tissue of Lean Mice. Cell Metab (2015) 21:543–57. doi: 10.1016/j.cmet.2015.03.005 PMC4747251 25863247

[89] Vasanthakumar A Moro K Xin A Liao Y Gloury R Kawamoto S. The Transcriptional Regulators IRF4, BATF and IL-33 Orchestrate Development and Maintenance of Adipose Tissue-Resident Regulatory T Cells. Nat Immunol (2015) 16:544–4. doi: 10.1038/ni0515-544d 25599561

[90] Han JM Wu D Denroche HC Yao Y Verchere B Levings MK. IL-33 Reverses an Obesity-Induced Deficit in Visceral Adipose Tissue ST2(+) T Regulatory Cells and Ameliorates Adipose Tissue Inflammation and Insulin Resistance. J Immunol (2015) 194:4777–83. doi: 10.4049/jimmunol.1500020 25870243

[91] Schiering C Krausgruber T Chomka A Fröhlich A Adelmann K Wohlfert EA. The Alarmin IL-33 Promotes Regulatory T-Cell Function in the Intestine. Nature (2014) 513:564. doi: 10.1038/nature13577 25043027 PMC4339042

[92] Feuerer M Herrero L Cipolletta D Naaz A Wong J Nayer A. Lean, But Not Obese, Fat is Enriched for a Unique Population of Regulatory T Cells That Affect Metabolic Parameters. Nat Med (2009) 15:930–U137. doi: 10.1038/nm.2002 19633656 PMC3115752

[93] Farini A Meregalli M Belicchi M Battistelli M Parolini D D’Antona G. T and B Lymphocyte Depletion has a Marked Effect on the Fibrosis of Dystronhic Skeletal Muscles in the Scid/Mdx Mouse. J Pathol (2007) 213:229–38. doi: 10.1002/path.2213 17668421

[94] Scharping NE Menk AV Moreci RS Whetstone RD Dadey RE Watkins SC. The Tumor Microenvironment Represses T Cell Mitochondrial Biogenesis to Drive Intratumoral T Cell Metabolic Insufficiency and Dysfunction. Immunity (2016) 45(2):374–88. doi: 10.1016/j.immuni.2016.07.009 PMC5207350 27496732

[95] Siska PJ Rathmell JC. T Cell Metabolic Fitness in Antitumor Immunity. Trends Immunol (2015) 36:257–64. doi: 10.1016/j.it.2015.02.007 PMC4393792 25773310

[96] Howie D Ten Bokum A Necula AS Cobbold SP Waldmann H. The Role of Lipid Metabolism in T Lymphocyte Differentiation and Survival. Front Immunol (2018) 8. doi: 10.3389/fimmu.2017.01949 PMC5770376 29375572

[97] Round JL Mazmanian SK. Inducible Foxp(3+) Regulatory T-Cell Development by a Commensal Bacterium of the Intestinal Microbiota. Proc Natl Acad Sci USA (2010) 107:12204–9. doi: 10.1073/pnas.0909122107 PMC2901479 20566854

[98] Atarashi K Tanoue T Shima T Imaoka A Kuwahara T Momose Y. Induction of Colonic Regulatory T Cells by Indigenous Clostridium Species. Science (2011) 331:337–41. doi: 10.1126/science.1198469 PMC3969237 21205640

[99] Smith PM Howitt MR Panikov N Michaud M Gallini CA Bohlooly-Y M. The Microbial Metabolites, Short-Chain Fatty Acids, Regulate Colonic T-Reg Cell Homeostasis. Science (2013) 341:569–73. doi: 10.1126/science.1241165 PMC3807819 23828891

[100] Tan J McKenzie C Vuillermin PJ Goverse G Vinuesa CG Mebius RE. Dietary Fiber and Bacterial SCFA Enhance Oral Tolerance and Protect Against Food Allergy Through Diverse Cellular Pathways. Cell Rep (2016) 15:2809–24. doi: 10.1016/j.celrep.2016.05.047 27332875

[101] Zheng Y Josefowicz S Chaudhry A Peng XP Forbush K Rudensky AY. Role of Conserved non-Coding DNA Elements in the Foxp3 Gene in Regulatory T-Cell Fate. Nature (2010) 463:808–U120. doi: 10.1038/nature08750 20072126 PMC2884187

[102] Stentz FB Kitabchi AE. Palmitic Acid-Induced Activation of Human T-Lymphocytes and Aortic Endothelial Cells With Production of Insulin Receptors, Reactive Oxygen Species, Cytokines, and Lipid Peroxidation. Biochem Biophys Res Commun (2006) 346:721–6. doi: 10.1016/j.bbrc.2006.05.159 16782068

[103] Szamel M Rehermann B Krebs B Kurrle R Resch K. Activation Signals In Human-Lymphocytes - Incorporation Of Poly-Unsaturated Fatty-Acids Into Plasma-Membrane Phospholipids Regulates Il-2 Synthesis Via Sustained Activation Of Protein Kinase-C. J Immunol (1989) 143(9):2806–13. 2530278

[104] Carrillo C Cavia MD Alonso-Torre S. Role of Oleic Acid in Immune System; Mechanism of Action; A Review. Nutricion Hospitalaria (2012) 27(4):978–90. https://doi.org/10.3305/nh.2012.27.4.5783 10.3305/nh.2012.27.4.5783 23165533

[105] Angela M Endo Y Asou HK Yamamoto T Tumes DJ Tokuyama H. Fatty Acid Metabolic Reprogramming *via* mTOR-Mediated Inductions of PPAR Gamma Directs Early Activation of T Cells. Nat Commun (2016) 7:13683. doi: 10.1038/ncomms13683 27901044 PMC5141517

[106] Palomer X Barroso E Zarei M Botteri G Vazquez-Carrera M. PPAR Beta/Delta and Lipid Metabolism in the Heart. Biochim Et Biophys Acta-Molecular Cell Biol Lipids (2016) 1861:1569–78. doi: 10.1016/j.bbalip.2016.01.019 26825692

[107] Kim W Barhoumi R McMurray DN Chapkin RS. Dietary Fish Oil and DHA Down-Regulate Antigen-Activated CD4+ T-Cells While Promoting the Formation of Liquid-Ordered Mesodomains. Br J Nutr (2014) 111(2):254–60. doi: 10.1017/S0007114513002444 PMC4327854 23962659

[108] Denys A Hichami A Khan NA. Eicosapentaenoic Acid and Docosahexaenoic Acid Modulate MAP Kinase Enzyme Activity in Human T-Cells. Mol Cell Biochem (2002) 232(1–2):143–8. doi: 10.1023/a:1014806122510 12030372

[109] Bonin A Khan NA. Regulation of Calcium Signalling by Docosahexaenoic Acid in Human T-Cells. Implication of CRAC Channels. J Lipid Res (2000) 41(2):277–4. 10681412

[110] Chow SC Jondal M. Polyunsaturated Free Fatty Acids Stimulate an Increase in Cytosolic Ca2+ by Mobilizing the Inositol 1,4,5-Trisphosphate-Sensitive Ca2+ Pool in T Cells Through a Mechanism Independent of Phosphoinositide Turnover. J Biol Chem (1990) 265(2):902–7. 2153118

[111] Aires V Hichami A Moutairou K Khan NA. Docosahexaenoic Acid and Other Fatty Acids Induce a Decrease in pHi in Jurkat T-Cells. Br J Pharmacol (2003) 140(7):1217–26. doi: 10.1038/sj.bjp.0705563 PMC1574148 14645139

[112] Terada S Takizawa M Yamamoto S Ezaki O Hiroshige I Akagawa KS. Suppressive Mechanisms of EPA on Human T Cell Proliferation. Microbiol Immunol (2001) 45(6):473–81. doi: 10.1111/j.1348-0421.2001.tb02647.x 11497223

[113] Zurier RB Rosetti RG Laposata M. Human Peripheral Blood T Lymphocyte Proliferation After Activation of the T Cell Receptor: Effects of Unsaturated Fatty Acids. Prostaglandins Leukotrienes Essential Fatty Acids (1999) 60(5–6):371–5. doi: 10.1016/s0952-3278(99)80015-5 10471124

[114] Radzikowska U Rinaldi AO Sözener ZC Karaguzel D Wojcik M Cypryk K. The Influence of Dietary Fatty Acids on Immune Responses. Nutrients (2019) 11(12):2990. doi: 10.3390/nu11122990 PMC6950146 31817726

[115] Mauro C Smith J Cucchi D Coe D Fu H Bonacina F. Obesity-Induced Metabolic Stress Leads to Biased Effector Memory CD4(+) T Cell Differentiation *via* PI3K p110 delta-Akt-Mediated Signals. Cell Metab (2017) 25:593–609. doi: 10.1016/j.cmet.2017.01.008 28190771 PMC5355363

[116] Zhou T Wang G Lyu Y Wang L Zuo S Zuo J. Upregulation of SLAMF3 on Human T Cells is Induced by Palmitic Acid Through the STAT5-PI3K/Akt Pathway and Features the Chronic Inflammatory Profiles of Type 2 Diabetes. Cell Death Dis (2019) 10(8):559. doi: 10.1038/s41419-019-1791-y 31332162 PMC6646391

[117] Hammer A Schliep A Jörg S Haghikia A Gold R Kleinewietfeld M. Impact of Combined Sodium Chloride and Saturated Long-Chain Fatty Acid Challenge on the Differentiation of T Helper Cells in Neuroinflammation. J Neuroinflamm (2017) 14:1–9. doi: 10.1186/s12974-017-0954-y PMC5596846 28899400

[118] Choi J-M Bothwell ALM. The Nuclear Receptor PPARs as Important Regulators of T-Cell Functions and Autoimmune Diseases. Mol Cells (2012) 33:217–22. doi: 10.1007/s10059-012-2297-y PMC3887706 22382683

[119] Yang XY Wang LH Chen T Hodge DR Resau JH DaSilva L. Activation of Human T Lymphocytes is Inhibited by Peroxisome Proliferator-Activated Receptor Gamma (PPAR Gamma) Agonists - PPAR Gamma Co-Association With Transcription Factor NFAT. J Biol Chem (2000) 275:4541–4. doi: 10.1074/jbc.275.7.4541 10671476

[120] Clark RB Bishop-Bailey D Estrada-Hernandez T Hla T Puddington L Padula SJ. The Nuclear Receptor PPAR Gamma and Immunoregulation: PPAR Gamma Mediates Inhibition of Helper T Cell Responses. J Immunol (2000) 164:1364–71. doi: 10.4049/jimmunol.164.3.1364 10640751

[121] Wohlfert EA Nichols FC Nevins E Clark RB. Peroxisome Proliferator-Activated Receptor Gamma (PPAR Gamma) and Immunoregulation: Enhancement of Regulatory T Cells Through PPAR Gamma-Dependent and -Independent Mechanisms. J Immunol (2007) 178:4129–35. doi: 10.4049/jimmunol.178.7.4129 17371968

[122] Su CG Wen X Bailey ST Jiang W Rangwala SM Keilbaugh SA. A Novel Therapy for Colitis Utilizing PPAR-Gamma Ligands to Inhibit the Epithelial Inflammatory Response. J Clin Invest (1999) 104:383–9. doi: 10.1172/JCI7145 PMC408529 10449430

[123] Desreumaux P Dubuquoy L Nutten S Peuchmaur M Englaro W Schoonjans K. Attenuation of Colon Inflammation Through Activators of the Retinoid X Receptor (RXR)/peroxisome Proliferator-Activated Receptor Gamma (BPAR Gamma) Heterodimer: A Basis for New Therapeutic Strategies. J Exp Med (2001) 193:827–38. doi: 10.1084/jem.193.7.827 PMC2193371 11283155

[124] Klotz L Burgdorf S Dani I Saijo K Flossdorf J Hucke S. The Nuclear Receptor PPAR Gamma Selectively Inhibits Th17 Differentiation in a T Cell-Intrinsic Fashion and Suppresses CNS Autoimmunity. J Exp Med (2009) 206:2079–89. doi: 10.1084/jem.20082771 PMC2757877 19737866

[125] Gocke AR Hussain RZ Yang Y Peng H Weiner J Ben L-H. Transcriptional Modulation of the Immune Response by Peroxisome Proliferator-Activated Receptor-Alpha Agonists in Autoimmune Disease. J Immunol (2009) 182:4479–87. doi: 10.4049/jimmunol.0713927 PMC2959196 19299749

[126] Polak PE Kalinin S Russo CD Gavrilyuk V Sharp A Peters JM. Protective Effects of a Peroxisome Proliferator-Activated Receptor-Beta/Delta Agonist in Experimental Autoimmune Encephalomyelitis. J Neuroimmunol (2005) 168:65–75. doi: 10.1016/j.jneuroim.2005.07.006 16098614

[127] Hua XX Wu J Goldstein JL Brown MS Hobbs HH. Structure Of The Human Gene Encoding Sterol Regulatory Element-Binding Protein-1 (Srebf1) And Localization Of Srebf1 And Srebf2 To Chromosomes 17p11.2 And 22q13. Genomics (1995) 25:667–73. doi: 10.1016/0888-7543(95)80009-B 7759101

[128] Kidani Y Elsaesser H Hock MB Vergnes L Williams KJ Argus JP. Sterol Regulatory Element-Binding Proteins are Essential for the Metabolic Programming of Effector T Cells and Adaptive Immunity. Nat Immunol (2013) 14:489. doi: 10.1038/ni.2570 23563690 PMC3652626

[129] Tauhid S Neema M Healy BC Weiner HL Bakshi R. MRI Phenotypes Based on Cerebral Lesions and Atrophy in Patients With Multiple Sclerosis. J Neurol Sci (2014) 346(1–2):250–4. doi: 10.1016/j.jns.2014.08.047 25220114

[130] Fletcher JM Lalor SJ Sweeney CM Tubridy N Mills KHG. T Cells in Multiple Sclerosis and Experimental Autoimmune Encephalomyelitis. Clin Exp Immunol (2010) 162(1):1–11. doi: 10.1111/j.1365-2249.2010.04143.x 20682002 PMC2990924

[131] Frischer JM Bramow S Dal-Bianco A Lucchinetti CF Rauschka H Schmidbauer M. The Relation Between Inflammation and Neurodegeneration in Multiple Sclerosis Brains. Brain (2021) 132:1175–89. doi: 10.1093/brain/awp070 PMC2677799 19339255

[132] Zhang J Weiner HL Hafler DA. Autoreactive T Cells in Multiple Sclerosis. Int Rev Immunol (1992) 9(3):183–201. doi: 10.3109/08830189209061790 1285060

[133] Zhang J Markovic-Plese S Lacet B Raus J Weiner HL Hafler DA. Increased Frequency of Interleukin 2-Responsive T Cells Specific for Myelin Basic Protein and Proteolipid Protein in Peripheral Blood and Cerebrospinal Fluid of Patients With Multiple Sclerosis. J Exp Med (1994) 179(3):973–84. doi: 10.1084/jem.179.3.973 PMC2191414 7509366

[134] Raine CS. The Dale E. McFarlin Memorial Lecture: The Immunology of the Multiple Sclerosis Lesion. Ann Neurol (1994) 36 Suppl:S61–72. doi: 10.1002/ana.410360716 8017891

[135] Peeters LM Vanheusden M Somers V Wijmeersch BV Stinissen P Broux Bieke. Cytotoxic CD4+ T Cells Drive Multiple Sclerosis Progression. Front Immunol (2017) 8. doi: 10.3389/fimmu.2017.01160 PMC5611397 28979263

[136] Fletcher JM Lalor SJ Sweeney CM Tubridy N Mills KHG. Roles of CD4 and CD8 T Lymphocytes in Multiple Sclerosis and Experimental Autoimmune Encephalomyelitis - Neuroinflammation - Wiley Online Library. Clin Exp Immunol (2010) 162(1):1-11. 20682002 10.1111/j.1365-2249.2010.04143.x PMC2990924

[137] Kaskow BJ Baecher-Allan C. Effector T Cells in Multiple Sclerosis. Cold Spring Harbor Perspect Med (2018) 8(4):a029025. doi: 10.1101/cshperspect.a029025 PMC5880159 29358315

[138] Linterman MA Beaton L Yu D Ramiscal RR Srivastava M Hogan JJ. IL-21 Acts Directly on B Cells to Regulate Bcl-6 Expression and Germinal Center Responses. J Exp Med (2010) 207(2):353–63. doi: 10.1084/jem.20091738 PMC2822609 20142429

[139] Batten M Ramamoorthi N Kljavin NM Ma CS Cox JH Dengler HS. IL-27 Supports Germinal Center Function by Enhancing IL-21 Production and the Function of T Follicular Helper Cells. J Exp Med (2010) 207(13):2895–906. doi: 10.1084/jem.20100064 PMC3005229 21098093

[140] Tzartos JS Craner MJ Friese MA Jakobsen KB Newcombe J Esiri MM. IL-21 and IL-21 Receptor Expression in Lymphocytes and Neurons in Multiple Sclerosis Brain. Am J Pathol (2011) 178(2):794–802. doi: 10.1016/j.ajpath.2010.10.043 21281812 PMC3032888

[141] Romme CJ Börnsen L Ratzer R Piehl F Khademi M Olsson T. Systemic Inflammation in Progressive Multiple Sclerosis Involves Follicular T-Helper, Th17- and Activated B-Cells and Correlates With Progression. PloS One (2013) 8(3):e57820. doi: 10.1371/journal.pone.0057820 23469245 PMC3585852

[142] Morita R Schmitt N Bentebibel SA Ranganathan R Bourdery L Zurawski G. Human Blood CXCR5(+)CD4(+) T Cells are Counterparts of T Follicular Cells and Contain Specific Subsets That Differentially Support Antibody Secretion. Immunity (2011) 34(1):108–21. doi: 10.1016/j.immuni.2010.12.012 PMC3046815 21215658

[143] Brucklacher-Waldert V Stuerner K Kolster M Wolthausen J Tolosa E. Phenotypical and Functional Characterization of T Helper 17 Cells in Multiple Sclerosis. Brain J Neurol (2009) 132(Pt 12):3329–41. doi: 10.1093/brain/awp289 19933767

[144] Frisullo G Nociti N Iorio R Patanella AK Marti A Caggiula M. IL17 and IFNgamma Production by Peripheral Blood Mononuclear Cells From Clinically Isolated Syndrome to Secondary Progressive Multiple Sclerosis. Cytokine (2008) 44(1):22–. doi: 10.1016/j.cyto.2008.08.007 18793860

[145] Leung S Liu X Fang L Chen X Guo T Zhang J. The Cytokine Milieu in the Interplay of Pathogenic Th1/Th17 Cells and Regulatory T Cells in Autoimmune Disease. Cell Mol Immunol (2010) 7(3):182–9. doi: 10.1038/cmi.2010.22 PMC4002916 20383174

[146] Harbour SN Maynard CL Zindl CL Schoeb TR Weaver CT. Th17 Cells Give Rise to Th1 Cells That are Required for the Pathogenesis of Colitis. Proc Natl Acad Sci USA (2015) 112(22):7061–6. 10.1073/pnas.1415675112 PMC4460486 26038559

[147] Uchiyama R Yonehara S Taniguchi S Ishido S Ishii KJ Tsutsui H. Inflammasome and Fas-Mediated IL-1β Contributes to Th17/Th1 Cell Induction in Pathogenic Bacterial Infection *In Vivo* . J Immunol (2017) 199(3):1122–30. doi: 10.4049/jimmunol.1601373 28674179

[148] Verstappen GM Corneth OBJ Bootsma H Kroese FGM. Th17 Cells in Primary Sjogren ‘ s Syndrome: Pathogenicity and Plasticity. J Autoimmun (2018) 87:16–25. doi: 10.1016/j.jaut.2017.11.003 29191572

[149] Kebir H Ifergan I Alvarez JI Bernard M Poirier J Arbour N. Preferential Recruitment of Interferon-Gamma-Expressing TH17 Cells in Multiple Sclerosis. Ann Neurol (2009) 66(3):390–402. doi: 10.1002/ana.21748. 19810097

[150] Domingues HS Mues M Lassmann H Wekerle H Krishnamoorthy G. Functional and Pathogenic Differences of Th1 and Th17 Cells in Experimental Autoimmune Encephalomyelitis. PloS One (2010) 5(11):e15531. doi: 10.1371/journal.pone.0015531 21209700 PMC3000428

[151] Dos Passos GR Sato DK Becker J Fujihara K. Th17 Cells Pathways in Multiple Sclerosis and Neuromyelitis Optica Spectrum Disorders: Pathophysiological and Therapeutic Implications. Mediators Inflammation (2016) 2016:5314541. doi: 10.1155/2016/5314541 PMC4749822 26941483

[152] Sie C Korn T Mitsdoerffer M. Th17 Cells in Central Nervous System Autoimmunity. Exp Neurol (2014) 262 Pt A. doi: 10.1016/j.expneurol.2014.03.009 24681001

[153] Martin F Apetoh L Ghiringhelli F. Controversies on the Role of Th17 in Cancer: A TGF-β-Dependent Immunosuppressive Activity? Trends Mol Med (2012) 18. doi: 10.1016/j.molmed.2012.09.007 23083809

[154] Zielinski CE Mele F Aschenbrenner D Jarrossay D Ronchi F Gattorno M. Pathogen-Induced Human T(H)17 Cells Produce IFN-Gamma or IL-10 and are Regulated by IL-1 Beta. Nature (2012) 484(7395):514–518. https://doi.org/10.1038/nature10957. 22466287 10.1038/nature10957

[155] Ustun C Miller JS Munn DH Weisdorf DJ Blazar BR. Regulatory T Cells in Acute Myelogenous Leukemia: Is it Time for Immunomodulation? Blood (2011) 118. doi: 10.1182/blood-2011-07-365817 PMC3217399 21881045

[156] Ye J Livergood RS Peng G. The Role and Regulation of Human Th17 Cells in Tumor Immunity. Am J Pathol (2013) 182(1):10–20. doi: 10.1016/j.ajpath.2012.08.041 23159950 PMC3532708

[157] Dominguez-Villar M Baecher-Allan CM Hafler DA. Identification of T Helper Type 1-Like, Foxp3+ Regulatory T Cells in Human Autoimmune Disease. Nat Med (2011) 17:673–5. doi: 10.1038/nm.2389 PMC3675886 21540856

[158] McClymont SA Putnam AL Lee MR Esensten JH Liu W Hulme MA. Plasticity of Human Regulatory T Cells in Healthy Subjects and Patients With Type 1 Diabetes. J Immunol (2011) 186:3918–26. doi: 10.4049/jimmunol.1003099 PMC3091943 21368230

[159] Kitz A de Marcken M Gautron AS Mitrovic MM Hafler DA Dominguez-Villar M. AKT Isoforms Modulate Th1-Like Treg Generation and Function in Human Autoimmune Disease. EMBO Rep (2016) 17:1169–83. doi: 10.15252/embr.201541905 PMC4967959 27312110

[160] Kitz A Dominguez-Villar M. Molecular Mechanisms Underlying Th1-Like Treg Generation and Function. Cell Mol Life Sci (2017) 74:4059–75. doi: 10.1007/s00018-017-2569-y PMC7079789 28624966

[161] de Carvalho JF Pereira RM Shoenfeld Y. The Mosiac of Autoimmunity: The Role of Environmental Factors. Front Biosci (2009) 1(2):501–9. https://doi.org/10.2741/e46 10.2741/e46 19482664

[162] Munz C Lunemann JD Getts MT Miller SD. Antiviral Immune Responses: Triggers of or Triggered by Autoimmunity? Nat Rev Immunol (2009) 9:246–58. doi: 10.1038/nri2527 PMC2854652 19319143

[163] Wells Hodans F Jean C Buzby. Dietary Assessment of Major Trends in U.S. Food Consumption, 1970-2005. Economic Information Bulletin No. 33. Economic Research Service, U.S. Dept. of Agriculture. March 2008.

[164] Hedstrom AK Olsson T Alfredsson L. High Body Mass Index Before Age 20 is Associated With Increased Risk for Multiple Sclerosis in Both Men and Women. Mult Scler (2012) 18:1334–6. doi: 10.1177/1352458512436596 22328681

[165] Munger KL Chitnis T Ascherio A. Body Size and Risk of MS in Two Cohorts of US Women. Neurology (2009) 73:1543–50. doi: 10.1212/WNL.0b013e3181c0d6e0 PMC2777074 19901245

[166] Guma M Tiziani S Firestein GS. Metabolomics in Rheumatic Diseases: Desperately Seeking Biomarkers. Nat Rev Rheumatol (2016) 12:269–81. doi: 10.1038/nrrheum.2016.1 PMC4963238 26935283

[167] Nogueras L Gonzalo H Jové́ M Sol J Gil-Sanchez A Hervá́s JV. Lipid Profile of Cerebrospinal Fluid in Multiple Sclerosis Patients: A Potential Tool for Diagnosis. Sci Rep (2019) 9:1–9. doi: 10.1038/s41598-019-47906-x 31383928 PMC6683197

[168] Lewkowicz N Piątek P Namiecińska M Domowicz M Bonikowski R Szemraji J. Naturally Occurring Nervonic Acid Ester Improves Myelin Synthesis by Human Oligodendrocytes. Cells (2019) 8. doi: 10.3390/cells8080786 PMC6721595 31362382

[169] Ferreira HB Melo T Monteiro A Paiva A Domingues P Domingues MR. Serum Phospholipidomics Reveals Altered Lipid Profile and Promising Biomarkers in Multiple Sclerosis. Arch Biochem Biophys (2021) 697:108672. doi: 10.1016/j.abb.2020.108672 33189653

[170] Chung Y Chang SH Martinez GJ Yang XO Nurieva R Kang HS. Critical Regulation of Early Th17 Cell Differentiation by Interleukin-1 Signaling. Immunity (2009) 30:576–87. doi: 10.1016/j.immuni.2009.02.007 PMC2705871 19362022

[171] Ghoreschi K Laurence A Yang X-P Tato CM McGeachy MJ Konkel JE. Generation of Pathogenic T(H)17 Cells in the Absence of TGF-Beta Signalling. Nature (2010) 467(7318):967–971. https://doi.org/10.1038/nature09447 20962846 10.1038/nature09447 PMC3108066

[172] Heink S Yogev N Garbers C Herwerth M Aly L Gasperi C. Trans-Presentation of IL-6 by Dendritic Cells is Required for the Priming of Pathogenic T(H)17 Cells. Nat Immunol (2017) 18:74–85. doi: 10.1038/ni.3632 27893700 PMC5164931

[173] Jain R Chen Y Kanno Y Joyce-Shaikh B Vahedi G Hirahara K. Interleukin-23-Induced Transcription Factor Blimp-1 Promotes Pathogenicity of T Helper 17 Cells. Immunity (2016) 44:131–42. doi: 10.1016/j.immuni.2015.11.009 PMC11608061 26750311

[174] Lee Y Awasthi A Yosef N Quintana FJ Xiao S Peters A. Induction and Molecular Signature of Pathogenic T(H)17 Cells. Nat Immunol (2012) 13:991–9. doi: 10.1038/ni.2416 PMC3459594 22961052

[175] Mailer RKW Joly AL Liu S Elias S Tegner J Andersson J. IL-1 Beta Promotes Th17 Differentiation by Inducing Alternative Splicing of FOXP3. Sci Rep (2015) 5. doi: 10.1038/srep14674 PMC4593960 26441347

[176] Pfeifle R Rothe T Ipseiz N Scherer HU Culemann S Harre U. Regulation of Autoantibody Activity by the IL-23-T(H)17 Axis Determines the Onset of Autoimmune Disease. Nat Immunol (2017) 18:104–13. doi: 10.1038/ni.3579 PMC5164937 27820809

[177] La Rocca C Carbone F De Rosa V Colamatteo A Galgani M Perna F. Immunometabolic Profiling of T Cells From Patients With Relapsing-Remitting Multiple Sclerosis Reveals an Impairment in Glycolysis and Mitochondrial Respiration. Metabol: Clin Exp (2017) 77:39–46. doi: 10.1016/j.metabol.2017.08.011 PMC5800394 29132538

[178] Jacobs SR Herman CE Maciver NJ Wofford JA Wieman HL Hammen JJ. Glucose Uptake is Limiting in T Cell Activation and Requires CD28-Mediated Akt-Dependent and Independent Pathways. J Immunol (2008) 180:4476–86. doi: 10.4049/jimmunol.180.7.4476 PMC2593791 18354169

[179] Yin Y Choi SC Xu Z Perry DJ Seay H Crocker BP. Normalization of CD4(+) T Cell Metabolism Reverses Lupus. Sci Trans Med (2015) 7. doi: 10.1126/scitranslmed.aaa0835 PMC5292723 25673763

[180] Van de KC Killestein J Popescu V Rijkers E Vrenken H Lutjohann D. Oxysterols and Cholesterol Precursors Correlate to Magnetic Resonance Imaging Measures of Neurodegeneration in Multiple Sclerosis. Multiple Sclerosis (Houndmills Basingstoke England) (2014) 20(4):412–7. doi: 10.1177/1352458513499421 23959711

[181] Uher T Fellows K Horakova D Zivadinov R Vaneckova M Sobisek L. Serum Lipid Profile Changes Predict Neurodegeneration in Interferon-β1a-Treated Multiple Sclerosis Patients. J Lipid Res (2017) 58(2):403–411. doi: 10.1194/jlr.M072751 27923871 PMC5282956

[182] Cantuti-Castelvetri L Fitzner D Bosch-Queralt M Weil MT Su M Sen P. Defective Cholesterol Clearance Limits Remyelination in the Aged Central Nervous System. Sci (New York NY) (2018) 359(6376):684–688. doi: 10.1126/science.aan4183 29301957

[183] Hubler Z Allimuthu D Bederman I Elitt MS Madhavan M Allan KC. Accumulation of 8,9-Unsaturated Sterols Drives Oligodendrocyte Formation and Remyelination. Nature (2018) 560(7718):372–376. doi: 10.1038/s41586-018-0360-3 30046109 PMC6423962

[184] Spann NJ Glass CK. Sterols and Oxysterols in Immune Cell Function. Nat Immunol (2013) 14(9):893–900. doi: 10.1038/ni.2681 23959186

[185] Lochner M Berod L Sparwasser T. Fatty Acid Metabolism in the Regulation of T Cell Function. Trends Immunol (2015) 36:81–91. doi: 10.1016/j.it.2014.12.005 25592731

[186] Grassi S Giussani P Mauri L Prioni S Sonnino S Prinetti A. Lipid Rafts and Neurodegeneration: Structural and Functional Roles in Physiologic Aging and Neurodegenerative Diseases. J Lipid Res (2020) 61(5):636–654. doi: 10.1194/jlr.TR119000427 31871065 PMC7193971

[187] Youssef S Stüve O Patarroyo JC Ruiz PJ Radosevich JL Hur EM. The HMG-CoA Reductase Inhibitor, Atorvastatin, Promotes a Th2 Bias and Reverses Paralysis in Central Nervous System Autoimmune Disease. Nature (2002) 420:78–84. doi: 10.1038/nature01158 12422218

[188] Zhang X Tao Y Troiani L Markovic-Plese S. Simvastatin Inhibits IFN Regulatory Factor 4 Expression and Th17 Cell Differentiation in CD4(+) T Cells Derived From Patients With Multiple Sclerosis. J Immunol (2011) 187:3431–7. doi: 10.4049/jimmunol.1100580 21856936

[189] Zhang X Tao Y Wang J Garcia-Mata R Markovic-Plese S. Simvastatin Inhibits Secretion of Th17-Polarizing Cytokines and Antigen Presentation by DCs in Patients With Relapsing Remitting Multiple Sclerosis. Eur J Immunol (2013) 43(1):281–9. doi: 10.1002/eji.201242566 23076801

[190] Xia M Hu S Fu Y Jin W Yi Q Matsui Y. CCR10 Regulates Balanced Maintenance and Function of Resident Regulatory and Effector T Cells to Promote Immune Homeostasis in Skin. J Allergy Clin Immunol (2014) 134:634–44. doi: 10.1016/j.jaci.2014.03.010 PMC4149943 24767879

[191] Burzyn D Kuswanto W Kolodin D Shadrach J Cerletti M Jang Y. A Special Population of Regulatory T Cells Potentiates Muscle Repair. Cell (2013) 155:1282–95. doi: 10.1016/j.cell.2013.10.054 PMC3894749 24315098

[192] Zeng H Chi H. mTOR and Lymphocyte Metabolism. Curr Opin Immunol (2013) 25:347–55. doi: 10.1016/j.coi.2013.05.002 PMC3759237 23722114

